# Physics-Based Device Models and Progress Review for Active Piezoelectric Semiconductor Devices

**DOI:** 10.3390/s20143872

**Published:** 2020-07-11

**Authors:** Hongseok Oh, Shadi A. Dayeh

**Affiliations:** Integrated Electronics and Biointerfaces Laboratory, Department of Electrical and Computer Engineering, University of California San Diego, La Jolla, CA 92093, USA; hoh@eng.ucsd.edu

**Keywords:** piezoelectric, sensor, transport model, nanowire, ZnO, Schottky diode, thin-film transistor (TFT)

## Abstract

Piezoelectric devices transduce mechanical energy to electrical energy by elastic deformation, which distorts local dipoles in crystalline materials. Amongst electromechanical sensors, piezoelectric devices are advantageous because of their scalability, light weight, low power consumption, and readily built-in amplification and ability for multiplexing, which are essential for wearables, medical devices, and robotics. This paper reviews recent progress in active piezoelectric devices. We classify these piezoelectric devices according to the material dimensionality and present physics-based device models to describe and quantify the piezoelectric response for one-dimensional nanowires, emerging two-dimensional materials, and three-dimensional thin films. Different transduction mechanisms and state-of-the-art devices for each type of material are reviewed. Perspectives on the future applications of active piezoelectric devices are discussed.

## 1. Introduction

Piezoelectric materials transduce mechanical energy to electrical energy by generating a voltage signal that is dependent on the applied stresses [1]. This transduction mechanism is derived from single-oriented internal dipoles. Under the presence of mechanical stimuli, these dipole moments are either strengthened or weakened depending on the direction and strength of the applied stress. Because they can be uniformly aligned, the change of each dipole moment collectively adds and generates a strong net dipole moment across the material [2]. This action generates two sheets of charges with opposite signs on the opposite surfaces of the material, usually in the direction of stress. Consequently, a variety of electromechanical sensors and transducers are engineered from these piezoelectric materials [3,4,5].

Ferroelectric materials, such as lead zirconate titanite (PZT), have been widely adopted owing to their high piezoelectric coefficient, which is a measure of a material’s ability to transduce mechanical forces to electric potentials. However, their application for electromechanical sensing is limited because they are electrically insulating [6]. This constraint also raises additional challenges for thin, lightweight, flexible, and stretchable electronics. Thus, conductive or semiconductive piezoelectric materials are advantageous because of their intrinsic amplification ability, such that small voltages generated by the piezoelectric effect can lead to large current changes. Depending on a device’s architecture, piezoelectric potentials can effectively and strongly modulate the charge transport behaviors, which can be easily measured to interpret stress. For instance, piezoelectric potentials can modulate the energy band profile at the contact interface and consequently alter the measured currents.

In this context, semiconductors with a non-centrosymmetric structure have received extensive research interest for electromechanical conversion due to their intrinsic piezoelectricity [7]. Binary crystals with structures lacking inversion symmetry, such as wurtzite or hexagonal crystals, possess internal piezoelectricity. Of these, zinc oxide (ZnO) and gallium nitride (GaN) are the most widely investigated materials for use in an array of electronic device architectures, such as thin-film transistors (TFTs) and high-electron mobility transistors (HEMT) [8,9,10]. A variety of growth methods can be used to prepare piezoelectric materials, including sputtering, sol–gel, metal–organic chemical vapor deposition (MOCVD), and pulsed laser deposition (PLD), resulting in diverse structures ranging from nanowires to thin films [11,12,13,14]. Films prepared with these techniques provide a wide spectrum of physical properties that can be tailored for different applications. In addition, previous work has focused on two-dimensional materials. The most pursued semiconducting two-dimensional materials, such as transition metal dichalcogenides, have a non-centrosymmetric crystal structure; thus, an effective piezoelectric response is expected [15]. To date, different electronic device architectures have been researched to utilize the effectively piezoelectric properties of different materials [16,17].

Dimensionality, similar to material morphology, can modulate a material’s piezoelectric response and can dictate the device’s structure and applications for electrochemical sensing. Therefore, we classify our discussions of piezoelectric materials into three main categories: nanowire, thin-film, and two-dimensional (2D) materials, as shown in Table 1. We will discuss each type in its respective section in this contribution, as detailed below.

## 2. Mechanism of Interaction between Piezoelectric Potential and Charge Transport

### 2.1. Generation of Polarization upon the Mechanical Stress

The generation of polarization field, $P_{i}$, in response to stress, $\sigma_{j}$, can be expressed as:

(1) $$P_{i}=d_{ij}\times \sigma_{j}=d_{ij}\times c_{jk}\times \epsilon_{k}\equiv e_{ik}\epsilon_{k}\;$$

where $d_{ij}$ is the piezoelectric moduli, $c_{jk}$ are elastic constants, $e_{ik}$ is the piezoelectric tensor, and $\epsilon_{k}$ is the strain tensor. For a wurtzite crystal with hexagonal symmetry and without shear, Equation (1) can be written in Voigt’s notation as a function of stress, as follows:

(2) $$\left(\begin{array}{c} P_{1}\\ P_{2}\\ P_{3} \end{array}\right)=\left(\begin{array}{cccccc} 0 & 0 & 0 & 0 & d_{15} & 0\\ 0 & 0 & 0 & d_{24} & 0 & 0\\ d_{31} & d_{31} & d_{33} & 0 & 0 & 0 \end{array}\right)\left(\begin{array}{c} \sigma_{1}\\ \sigma_{2}\\ \sigma_{3}\\ \sigma_{4}\\ \sigma_{5}\\ \sigma_{6} \end{array}\right)$$

Alternatively, it can be written as a function of strain:

(3) $$\left(\begin{array}{c} P_{1}\\ P_{2}\\ P_{3} \end{array}\right)=\left(\begin{array}{cccccc} 0 & 0 & 0 & 0 & e_{15} & 0\\ 0 & 0 & 0 & e_{24} & 0 & 0\\ e_{31} & e_{31} & e_{33} & 0 & 0 & 0 \end{array}\right)\left(\begin{array}{c} \epsilon_{1}\\ \epsilon_{2}\\ \epsilon_{3}\\ \epsilon_{4}\\ \epsilon_{5}\\ \epsilon_{6} \end{array}\right)$$

For stress applied only along the c-axis direction, the polarization charge density, $\sigma^{PZ}$, can be expressed as:

(4) $$\sigma^{PZ}=\left|P_{3}\right|=d_{33}\sigma_{3}=d_{33}F/A$$

where *F* is the applied force and *A* is the cross-sectional area. Consequently, the piezoelectric coefficient $d_{33}$ determines the material’s electronic response to stress. While PZT possesses a strong $d_{33}$ component of 374 pC/N [18], piezoelectric semiconductors such as ZnO and GaN have relatively low $d_{33}$ coefficients of 12.4 pC/N and 3.1 pC/N, respectively [19,20]. However, amplification strategies that are conveniently accomplished with semiconductor materials can mitigate this sensitivity gap. As such, the generated piezoelectric charges on the two faces of a semiconductor film can provide an exponential amplification in a device with a Schottky contact, or a linear amplification of current through the film in a field-effect transistor device.

The most prominent effect of the polarization charge density for all pressure levels and semiconductor doping densities is its impact on the space charge density near the device surface. We will first discuss this in the context of Schottky diodes and then in the context of field-effect transistors (FETs).

### 2.2. Schottky Diodes as Piezoelectric Sensors

**Figure 1:**
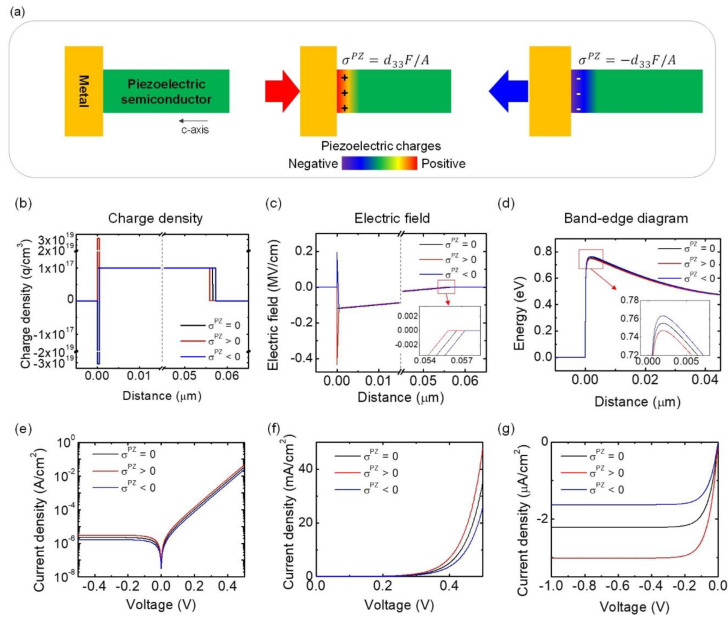
Mechanism of piezoelectric modulation in Schottky diodes. (**a**) Schematic illustration of the device with a Schottky contact and response under compressive ($\sigma^{PZ}>0$) or tensile stress $\left(\sigma^{PZ}<0\right)$. (**b**–**d**) Plots of (**b**) charge density, (**c**) electric field (inset: detailed plots of electric field near the end of depletion region) and (**d**) energy band edge diagram of the Schottky diode under compressive and tensile stress (inset: detailed plots at the peak of the band edge plots), respectively. (**e**–**g**) Current density vs. voltage characteristics with and without piezoelectric charges generated from the analytic solutions. (**e**) Semilog plot of current density vs. voltage curves. Linear plots of the curves with (**f**) forward bias and (**g**) reverse bias regimes. Black, red, and blue curves represent no stress, compressive stress, and tensile stress, respectively.

Two-terminal structures with one-sided or back-to-back Schottky contacts have been widely employed to make active piezoelectric devices due to their ease of fabrication. Figure 1a shows the typical structure of a rectifying Schottky metal–semiconductor contact. Positive or negative piezoelectric charges accumulate at the interface, depending on the direction of the stress and the polarity (anion or cation) at the interface plane (middle and right of Figure 1a).

The change of the energy band edge structure can be derived by using an abrupt junction model with a thin piezoelectric charge at the interface. We assume that the metal–semiconductor interface is located at x=0, the semiconductor has a uniform doping density $N_{D}$, and piezoelectric charge is distributed over $0<x<\delta_{s}$ with charge density of $\sigma^{PZ}/q\delta_{s}$, where $\delta_{s}$ is of the order of 1 nm or less. The equilibrium Schottky barrier height (SBH), built-in potential, and the applied bias are denoted by $\varphi_{Bn}^{0}$, $V_{bi}$, and V. The analytic solutions for charge density, electric field, and electric potential obtained by solving the 1D Poisson’s equation are summarized in Table 2. The detailed step-by-step derivations can be found in Appendix A1 and Table A1, Table A2 and Table A3).

These dependencies in Table 2 are portrayed in the numerical calculations in Figure 1b–d. To put these equations in perspective, we consider a ZnO-Au Schottky junction with applied pressure of 200 MPa, $q\varphi_{Bn}^{0}$ = 0.8 eV, *V_bi_* = 0.34 V, *V* = 0 V, *N_D_* = 1 × 10^17^cm^−3^, *σ^PZ^*/*q* = ± 1.55 × 10^12^ cm^−2^ (for different stress polarities), and $\delta_{s}$ = 0.5 nm. The full list of parameters can be found in the Appendix A1.3 Table A4 and Table A5). Figure 1b shows significant positive or negative piezoelectric charge located next to the interface, which in turn induces changes in the depletion width. While the equilibrium depletion width without piezoelectric charge is 56.6 nm, it reduced to 55.8 nm with *σ^PZ^* > 0 and increased to 57.2 nm with $\sigma^{PZ}<0$. Even though the change of the depletion width is small, this results in more than 30% of the current change in both reverse and forward biases. The piezoelectric charge induces a strong electric field of about 0.3 MV near the interface, as is depicted in Figure 1c. The electric field reduced to zero at the edge of the piezoelectrically modulated depletion width, as shown in the insets. Finally, Figure 1d shows the resulting energy band edge diagrams, which also accounts for lowering of the image force. Under equilibrium and without piezoelectric charge, the SBH is reduced by 45 meV to 0.755 eV due to the image force lowering. On the other hand, piezoelectric charge decreases or increases the SBH by 8 meV when $\sigma^{PZ}>0$ or $\sigma^{PZ}<0$, respectively, for F/A = 200MPa.

In the presence of piezoelectric polarization charge density, the depletion width in a Schottky diode can be expressed as:

(5) $$W_{D}=\sqrt{\frac{2\varepsilon_{s}\left(V_{bi}-V\right)}{qN_{D}}-\frac{\sigma^{PZ}\delta_{s}}{qN_{D}}\;}$$

Equation (5) reduces to the well-known depletion width equation $W_{D}=\sqrt{2\varepsilon_{s}\left(V_{bi}-V\right)/qN_{D}}$ in the absence of $\sigma^{PZ}$. For charge transport, modulation of $W_{D}$ with strain results in modulation of the tunneling component of the current density across the diode.

The change of SBH can be expressed as:

(6) $$\varphi_{Bn}^{\prime }=\varphi_{Bn}^{0}-\Delta \varphi_{Bn}^{IFL}+\Delta \varphi_{Bn}^{PZ}$$

where the Schottky barrier height equilibrium, $\varphi_{Bn}^{0}$, is expressed as:

(7) $$\varphi_{Bn}^{0}=c_{2}\left(\phi_{m}-\chi_{s}\right)+\left(1-c_{2}\right)\left(\frac{E_{g}}{q}-\varphi_{0}\right)$$

where $\phi_{m}$ is the metal work function, $\chi_{s}$ is the semiconductor electron affinity, *E_g_* is the energy bandgap, $\varphi_{0}$ is the charge neutrality level, *q* is the fundamental charge constant, *ε_s_* is the dielectric constant, and $c_{2}=\varepsilon_{0}/\left(\varepsilon_{0}+q^{2}\delta_{i}D_{s}\right)$. Here, $\varepsilon_{0}$ is the vacuum dielectric constant, $\delta_{i}\approx$ 0.5 nm, and $D_{s}$ is the surface state density per unit area and unit energy [21]. The image force lowering $\Delta \varphi_{Bn}^{IFL}$ is expressed as:

(8) $$\Delta \varphi_{Bn}^{IFL}={\left(\frac{q^{3}N_{D}\left|V_{bi}-V\right|}{8\pi^{2}\varepsilon_{s}^{3}}\right)}^{\frac{1}{4}}$$

The change of the SBH due to piezoelectric force, or $\Delta \varphi_{Bn}^{PZ}$, is expressed as:

(9) $$\Delta \varphi_{Bn}^{PZ}=-\frac{\sigma^{PZ}\delta_{s}}{2\varepsilon_{s}}$$

which is the shift in the barrier height due to the strain-induced polarization charge density.

The effective change of the SBH can be easily resolved by measuring the reverse-bias current. The current across the Schottky junction is expressed as:

(10) $$J\approx A^{**}T^{2}\exp\left(-\frac{q\varphi_{Bn}^{\prime }}{kT}\right)\left(\exp\left(\frac{V}{nkT}\right)-1\right)$$

Here, A^**^ is the effective Richardson constant, *T* is the temperature, $\varphi_{Bn}^{\prime }$ is the Schottky barrier height, *k* is the Boltzmann constant, and n is the ideality factor of this junction. Figure 1e shows the semilog plot of resulting current density vs. voltage with different piezoelectric charges. The positive piezoelectric charge will increase the current with both forward and reverse biases, while the negative piezoelectric charge exhibits the opposite pattern. Figure 1f shows the linear plot of current density for the forward bias, indicating that different piezoelectric charges result in different turn-on voltages. On the other hand, as shown in Figure 1g, the current density was constant under sufficient reverse bias and the shifts of the current level only depend on the piezoelectric charge. Under sufficient reverse bias conditions, the reverse saturation current density can be expressed as [21]:

(11) $$J_{R}\approx J_{0}\approx A^{**}T^{2}\exp\left(-\frac{q\varphi_{Bn}^{\prime }}{kT}\right)$$

The effective change in the Schottky barrier height leads to an exponential change in the current, enabling high sensitivity for relatively small piezoelectric charges, which as noted earlier is the advantage of semiconductor piezoelectric devices.

Many Schottky contact-based nanowire devices serve as strain sensors, which are surveyed in this work. The sensitivity of the device to the applied strain can be represented by the Gauge factor, which is prominently used to compare the performance of strain sensors [22]. Gauge factor G is defined as the change of current in the device normalized by the applied strain:

(12) $$G=\frac{1}{\epsilon_{3}}\left|\frac{\Delta I}{I_{0}}\right|\;$$

where $I_{0}$ is the baseline current without strain and ΔI is the current change due to the strain $\epsilon_{3}$. For a device under reverse bias, the current change due to the piezoelectric charge is:

(13) $$\frac{\Delta J_{R}}{J_{R}}\approx \exp\left(-\frac{q\Delta \varphi_{Bn}^{PZ}}{kT}\right)-1$$

On the other hand, the change of the SBH due to the piezoelectric charge considering only normal stress is:

(14) $$\Delta \varphi_{Bn}^{PZ}=-\frac{\sigma^{PZ}\delta_{s}}{2\varepsilon_{s}}=-\frac{\left(2e_{31}\epsilon_{1}+e_{33}\epsilon_{3}\right)\delta_{s}}{2\varepsilon_{s}}$$

where $e_{33}$ is the piezoelectric tensor component along the *c*-axis. Normalization by the applied stress gives the gauge factor of the device as:

(15) $$G=\frac{1}{\epsilon_{3}}\left|\frac{\Delta J_{R}}{J_{R}}\right|\approx \frac{1}{\epsilon_{3}}\left[\exp\left(\frac{q\left(2e_{31}\epsilon_{1}+e_{33}\epsilon_{3}\right)\delta_{s}}{2\varepsilon_{s}kT}\right)-1\right]$$

### 2.3. Metal–Insulator–Semiconductor (MIS) Thin-Film Transistors (TFTs) as Piezoelectric Sensors

**Figure 2:**
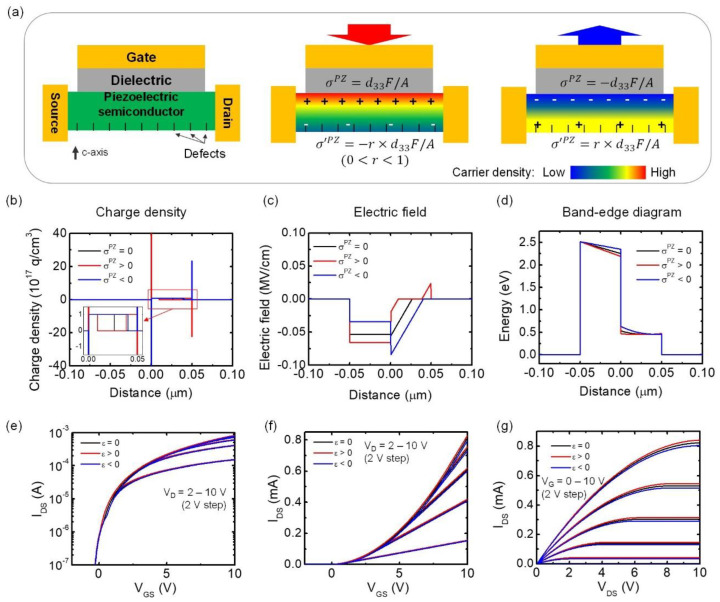
Piezoelectric MIS thin-film transistors (TFTs). (**a**) Schematic diagram of the device with compressive and tensile out-of-plane stress. (**b**–**d**) Plots of the (**b**) charge density (Inset: detailed charge density distributions inside the film region), (**c**) electric field, and (**d**) energy band edge across the gate, under compressive and tensile stress, respectively. (**e**–**g**) Transfer and output characteristics of the MIS TFTs with and without stress generated from the analytic solutions. (**e**) Transfer curves plotted in semilog scale and (**f**) the same curves plotted in linear scale. (**g**) Output curves. Black, red, and blue curves represent no stress, compressive stress, and tensile stress, respectively.

In piezoelectric sensors employing FETs, sheets with piezoelectric charges $\sigma^{PZ}$ and ${\sigma^{\prime }}^{PZ}$ are ideally created on the top and the bottom surfaces of the thin film, with equal charge densities and opposite signs (${\sigma^{\prime }}^{PZ}$ = − *σ^PZ^*). Here, we assume that under positive stress, the positive piezoelectric charge is created at the semiconductor–insulator interface facing the gate side, although the opposite can yield similar results with opposite polarity. The piezoelectric charge on the gate side alters the surface potential of the FET and modulates the width of depletion region, as well as the flat-band voltage. On the other hand, the piezoelectric charge on the back side of the film will deplete or accumulate charges. Therefore, it is reasonable to treat this charge modulation as a modulation of the effective thickness of the conductive channel. According to the principle of charge neutrality, the effects of two piezoelectric sheet charges at the front and the back surfaces of the film should cancel out each other. However, imperfections in film properties distort this ideal charge neutrality condition. In thin film growth, the nucleation layers are usually highly defective and are bound by grain boundaries that can terminate piezoelectric fields, which means ${\sigma^{\prime }}^{PZ}$ = − *rσ^PZ^*, where 0<r<1. (0 means all the piezoelectric charges are terminated, and 1 means there is no termination.) This reinforces the condition of piezoelectric charge balance on the back of the film. Considering this effect, we set the piezoelectric charge density on the back surface to a fraction (r = 1/2) of that on the front surface in this section. Figure 2a shows a schematic illustration of the change of carrier density inside the thin film under positive or negative stress.

To investigate the MIS TFT piezoelectric response, we solve the 1D Poisson’s equation to calculate the electric fields and potentials of the charge in the device. We assume that the metal–semiconductor interface is located at x=0, the gate insulator has a thickness of d (−d<x<0), the semiconductor has a thickness of $t_{s}$ and a uniform doping density $N_{D}$, and that the piezoelectric charge is distributed over $0<x<\delta_{s}$ and $t_{s}-\delta_{s}<x<t_{s}$, with charge density of $\sigma^{PZ}/q\delta_{s}$ and ${\sigma^{\prime }}^{PZ}/q\delta_{s}$, respectively, where $\delta_{s}$ is of the order of 1nm or less. The flat-band potential and the applied bias are denoted by $V_{FB}$ and V. The analytic solutions for the charge density, electric field, and electric potential derived from the 1D Poisson’s equation are summarized in Table 3. The detailed step-by-step derivations can be found in the Appendix A2.1 and Table A6, Table A7 and Table A8. These dependencies are portrayed in Figure 2b–d, where plots were generated using the equations in Table 3. We illustrate the phenomenological piezoelectric response in a ZnO TFT with applied pressure of 30 MPa, $V_{FB}$ = 0.34 eV, V = 0 V, $N_{D}$ = 1 × 10^17^cm^−3^, $\sigma^{PZ}/q$= ± 2.325 × 10^11^ cm^−2^,$\;\delta_{s}$ = 0.5, $t_{s}$ = 50 nm, and d = 50 nm. The full list of parameters can be found in Appendix A2.3 and Table A9.

Figure 2b shows strong positive or negative piezoelectric charges located on the front (gate electrode side) and back surfaces of the film under positive stress. Due to the presence of piezoelectric charges, the width of the depletion region near the gate electrode decreases. On the other hand, depletion of free charge carriers was induced on the back surface of the film due to the presence of negative piezoelectric charges. This depletion is denoted by the positive charge density from 0.034–0.05 μm in the red curve of Figure 2b and the inset. On the other hand, the blue curve of Figure 2b illustrates the charge distribution under negative stress. The negative piezoelectric charge on the front surface increased the depletion region. The effect of positive piezoelectric charge on the back surface is not plotted here, since the accumulated electron density on the back is expected to be very thin. Figure 2c shows the resulting electric field across the MIS structure. The piezoelectric charge causes a redistribution of the field inside the dielectric layer and the surface depletion region, while maintaining the potential drop across the structure. The resulting energy band edge plot is shown in Figure 2d, a depicting downward or upward band bending with positive or negative stress, respectively.

Importantly, the width of the depletion region on the front and back surfaces, denoted by $W_{D}$ and ${W_{D}}^{\prime }$, can be calculated as:

(16) $$W_{D}=-\frac{\varepsilon_{s}}{C_{i}}+\sqrt{\frac{2\varepsilon_{s}}{qN_{D}}\left(V_{FB}-V-\frac{\sigma^{PZ}}{C_{i}}-\frac{\sigma^{PZ}\delta_{s}}{2\varepsilon_{s}}\right)+{\left(\frac{\varepsilon_{s}}{C_{i}}\right)}^{2}}$$

and

(17) $$W_{D}^{\prime }=\frac{{\sigma^{\prime }}^{PZ}}{qN_{D}}\;\mathrm{for}\;{\sigma^{\prime }}^{PZ}<0\;\mathrm{and}\;W_{D}^{\prime }=0\;\mathrm{for}\;{\sigma^{\prime }}^{PZ}>0\;$$

Due to the bulk conduction in piezoelectric TFTs, these widths are important in the calculation of the total TFT current.

Once the charge distribution is known, we can calculate the current $I_{DS}$ for given gate and drain voltages, $V_{G}$ and $V_{D}$, respectively. However, it should be noted that piezoelectric TFTs are junctionless and operate in accumulation mode; the bulk segment of the TFT will also conduct a component of the current due to the abundance of unintentionally doped free carriers across the oxide layer. This necessitates the development of a specific set of current–voltage characteristic equations for piezoelectric TFTs. The current in the TFT can be divided into two components: (i) surface current $I_{s}$ via accumulated electrons with surface mobility of $\mu_{s}$ and (ii) a bulk current $I_{b}$ via intrinsic carriers with bulk mobility of $\mu_{b}$ [21]. The total current will be the sum of the bulk and surface currents:

(18) $$I_{DS}=I_{s}+I_{b}$$

In this model, the threshold voltage $V_{T}$ is defined when the entire film is depleted. The current starts to flow in the bulk of the film when $V_{G}>V_{T}$. The bulk current is maximized when $V_{G}=V_{FB}$ and there is no depletion region, while the surface current emerges when surface accumulation starts at $V_{G}>V_{FB}$. From these assumptions and gradual channel approximation, the equations for $I_{DS}$ are summarized in Table 4. The detailed step-by-step derivations can be found in Appendix A2.2.

Under the presence of the piezoelectric charge, the effective conduction layer thickness is changed, since the piezoelectric charge on the back surface either depletes or accumulates electrons in its vicinity. The effective thickness of the film $t_{s}^{PZ}$ is expressed as:

(19) $$t_{s}^{PZ}=t_{s}-\frac{{\sigma^{\prime }}^{PZ}}{qN_{D}}$$

For positive stress, $t_{s}^{PZ}$ decreases corresponding to the additional depletion region on the back surface of the film. For negative stress, $t_{s}^{PZ}$ increases to larger than $t_{s}$, resembling an increased electron charge density on the back surface. The threshold voltage can now be defined, where $W_{D}=t_{s}^{PZ}$, expressed as:

(20) $$V_{T}^{PZ}=V_{FB}-\frac{\sigma^{PZ}}{C_{i}}-\frac{\sigma^{PZ}\delta_{s}}{2\varepsilon_{s}}-\frac{qN_{D}}{2\varepsilon_{s}}\left\{{\left(t_{s}^{PZ}+\frac{\varepsilon_{s}}{C_{i}}\right)}^{2}-{\left(\frac{\varepsilon_{s}}{C_{i}}\right)}^{2}\right\}$$

where $C_{i}$ is the capacitance of the gate insulator.

The accumulation channel is created when $V_{G}>V_{FB}$. In the presence of piezoelectric charge, the flat-band voltage $V_{FB}^{PZ}$ can be expressed as:

(21) $$V_{FB}^{PZ}=V_{FB}-\frac{\sigma^{PZ}}{C_{i}}-\frac{\sigma^{PZ}\delta_{s}}{2\varepsilon_{s}}$$

from the condition that $V_{G}=V_{FB}$ when the fixed charge density in the TFT channel is zero and $W_{PZ}=0$. Given $t_{s}^{PZ}$, $V_{T}^{PZ}$, and $V_{FB}^{PZ}$, the equations in Table 4 can be used to calculate the current–voltage characteristics of the TFTs.

We generated the corresponding transfer and output curves using the equations in Table 4, as shown in Figure 2e–g. Figure 2e shows the semilog plot of transfer characteristics. The TFT could be turned on at ~ $V_{G}$ = −0.5 V, and almost no differences were observed for all stress cases. As expected from the above discussions, positive stress turns on the surface channel earlier, resulting in a higher current level. The linear plot of transfer characteristics in Figure 2f and output characteristics in Figure 2g show that the piezoelectric charge effects are larger at higher $I_{DS}$ values.

The sensitivity of the device can be obtained by dividing the relative current change by the applied stress. In the saturation regime, the current is expressed as:

(22) $$I_{DS}=\frac{Z}{L}\mu_{s}C_{i}\frac{{\left(V_{G}-V_{FB}\right)}^{2}}{2}+\frac{qN_{D}Z}{L}\mu_{b}t_{s}\left(V_{G}-V_{FB}+\frac{qN_{D}}{2C_{i}}t_{s}\right)=I_{s}+I_{b}$$

Under sufficient gate bias, accumulation channel current $I_{s}$ is much larger than the bulk current $I_{b}$. Hence, the sensitivity can be written as:

(23) $$S=\frac{\frac{\Delta I_{DS}}{I_{DS}}}{\sigma_{3}}\approx \frac{\frac{\Delta I_{s}}{I_{s}}}{\sigma_{3}}=\frac{\left(2\left(V_{G}-V_{FB}\right)+\frac{\sigma^{PZ}}{C_{i}}+\frac{\sigma^{PZ}\delta_{s}}{2\varepsilon_{s}}\right)\left(\frac{\sigma^{PZ}}{C_{i}}+\frac{\sigma^{PZ}\delta_{s}}{2\varepsilon_{s}}\right)}{\sigma_{3}{\left(V_{G}-V_{FB}\right)}^{2}}\;≅\frac{2d_{33}}{C_{i}\left(V_{G}-V_{FB}\right)}+\frac{d_{33}^{2}\sigma_{3}}{C_{i}^{2}{\left(V_{G}-V_{FB}\right)}^{2}}≅\frac{2d_{33}}{C_{i}\left(V_{G}-V_{FB}\right)}$$

$$(\mathrm{Note}\;\mathrm{that}\;\sigma^{PZ}=d_{33}\sigma_{3},\;\frac{\sigma^{PZ}\delta_{s}}{2\varepsilon_{s}}\ll \frac{\sigma^{PZ}}{C_{i}}\;\mathrm{and}\;\frac{d_{33}^{2}\sigma_{3}}{C_{i}^{2}}≅0).$$

## 3. Nanowire-Based Piezoelectric Devices

### 3.1. Piezoelectric Nanowire-Based Strain Sensors

The advent of nanowire (NW) research in the early 2000′s inspired the development of novel nanowire-based piezoelectric devices. Among many types of nanowires, ZnO—one of the extensively pursued materials for nanowires—has several advantages for piezoelectric device applications. It has a strong piezoelectric coefficient of 12.4 pC/N compared to other semiconductors. Moreover, its c-axis is parallel to the axial (growth) direction. Therefore, the piezoelectric charges can be easily accessed by placing electrodes on each end of the nanowires. Importantly, ZnO nanowires can be easily synthesized using the vapor–liquid–solid (VLS) growth mechanism, either by employing metal catalysts [23] or by catalyst-free growth mechanisms [24]. Techniques used for growth generally include chemical vapor deposition [25], hydrothermal growth [26], solution growth techniques [27], and metal organic vapor phase epitaxy [28]. Thanks to their structural advantages and versatile synthesis methods, the use of ZnO nanowire-based piezoelectric devices became popular in strain and force sensors.

**Figure 3:**
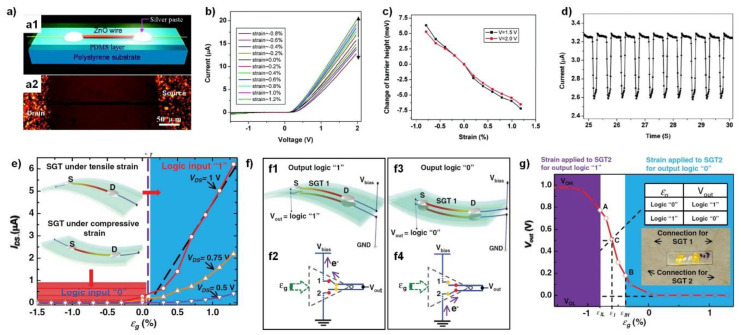
Nanowire-based piezoelectric electromechanical sensors. (**a**–**d**) Flexible piezoelectric strain sensor. (**a**) Structure of the ZnO piezoelectric fine wire (PFW) strain sensor. (**a**1) Schematic illustration and (**a**2) microscope image of the strain sensor using a ZnO PFW. (**b**) The I-V characteristics of the device under different strains. (**c**) Calculated change of Schottky barrier height as a function of strain. (**d**) Temporal response curves under stimuli of 2 Hz. (**a**–**d**) Reproduced from [29] with permission from the American Chemical Society. Copyright 2008. (**e**–**g**) Strain-gated piezoelectric logic nanodevices. (**e**) The *I_DS_–ε_g_* strain–voltage transfer characteristics of the ZnO strain-gated transistor (SGT). (**f**) Schematics of the ZnO SGT-based inverter (SGI) performing logic operations in the responding input strain. (**f**1) When the strain input logic is “0”, the output signal logic is “1”. (**f**2) Schematic diagram showing that SGT 1 is on and SGT 2 is off (**f**3). (**f**1) When the strain input logic is “1”, the output signal logic is “0”. (**f**4) Schematic diagram showing that SGT 1 is off and SGT 2 is on. (**g**) Resulting strain–voltage transfer characteristics. (**e**–**g**) Reproduced from [30] with permission from Wiley-VCH. Copyright 2010.

The typical structure of ZnO nanowire-based piezoelectric devices is illustrated in the work by Zhou et al. [29]. Here, ZnO piezoelectric fine wire (PFW)—a thicker version of nanowires with diameters ranging 2–6 μm and lengths of several hundred micrometers to several millimeters—was dispersed on a flexible polystyrene substrate and two electrodes were fabricated at each end of the nanowire (Figure 3a). In this device, as shown in Figure 3b, asymmetric current-voltage (I-V) characteristics were achieved due to the different SBHs at each metal–ZnO junction. Compressive or tensile strain was applied to the ZnO PFW by bending the substrate. The strain induces piezoelectric charges and changes the SBH (Figure 3c), as discussed in Chapter 2, and leads to the large current modulation in proportion to the strain. The device exhibited a fast response of ~10 ms, as shown in Figure 3d, and a high gauge factor of up to 1250. The approach of fabricating two-terminal nanowire devices using dispersed nanowires on flexible substrates was then widely adopted due to the simple strain sensing mechanism involved.

One of the interesting applications for ZnO piezoelectrics was the strain-gated logic devices made by Wu et al., where tensile–compressive strain played the role of a gate electrode in a conventional field effect transistor (Figure 3e–g) [30]. The device was optimized to show large current modulation under the presence of strain, which allowed strain-induced current switching. Figure 3e shows the typical strain–voltage transfer characteristics, which were obtained by plotting the current as a function of corresponding strains. The device achieved an *I_on_/I_off_* ratio of 112 at $V_{DS}$ = 1 V when the strain varied in the range of 1%. This property was named the strain-gated transistor (SGT). Various types of logic gates were constructed using these SGTs, including inverter (Figure 3f), NAND, and NOR logic gates, and the noise margin was analyzed according to the strain–voltage transfer characteristics (Figure 3g). Here, the combination of input strain values for two SGTs generated electrical outputs of either 0 or 1.

### 3.2. Vertical Nanowire-Based Strain and Force Sensors

**Figure 4:**
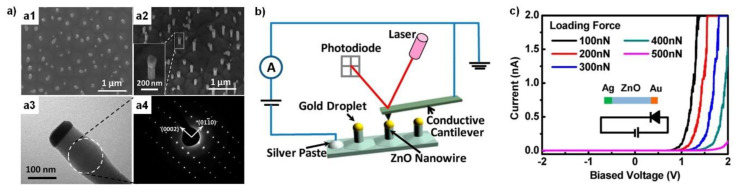
(**a**–**c**) ZnO vertical nanowires and investigation of their strain response. (**a**) Morphology and structural analysis of the vertically grown ZnO nanowires. (**a**1,**a**2) Tilted-view SEM images, along with (**a**3,**a**4) typical TEM image and corresponding selected area electron diffraction (SAED) image of the ZnO nanowire with gold tip. (**b**) Illustration of the measurement setup. (**c**) I-V characteristics of the device under different downward forces. (**a**–**c**) Reproduced from [32] with permission from the American Chemical Society. Copyright 2012.

Despite the advantages of the lateral nanowire devices discussed above, the randomness of dispersed nanowires has remained a critical drawback for practical applications. In fact, most nanowires grow vertically during synthesis [11,19,31]. Hence, it is natural to fabricate devices using vertical nanowires. Han et al. investigated the piezoelectric response of vertical ZnO nanowires that were heteroepitaxially grown on GaN thin films [32]. Shown in Figure 4a are the morphology and microstructure of the grown ZnO nanowires, containing gold catalyst droplets on the tips of the vertical nanowires. The piezoelectric response of the nanowires was measured using a conductive cantilever, as illustrated in Figure 4b. The silver paste on the GaN thin film and the gold droplet served as the cathode and the anode of the test device, respectively. The I-V curves in Figure 4c clearly show the force-dependent current modulation, manifesting the force-sensing ability from the vertically grown nanowires.

The scalable fabrication of the nanowire-based strain sensor array was achieved by Wu et al. [33]. The authors fabricated a tactile pixel (taxel) array, which was composed of a 92 × 92 passive-matrix vertical ZnO nanowire two-terminal device. Here, vertically aligned ZnO NWs were grown selectively on gold electrodes using a hydrothermal method, followed by casting of the polymer SU-8 as an insulating spacer layer between the top and bottom electrodes. The ZnO NWs were sandwiched between two gold electrodes and formed back-to-back Schottky contacts. When an external force was applied, the Schottky barrier characteristics changed in response and the transport behavior across the ZnO nanowire channel was modulated in accordance with the external stress. By recording the current changes of each element in this 92 × 92 taxel array and generating heatmaps, the distribution of the applied force on the array was well visualized. Owing to its flexible substrate, the array functioned even when it was flexed. The research on nanowire-based strain sensors is summarized in Table 5.

### 3.3. Piezophototronic Devices

**Figure 5:**
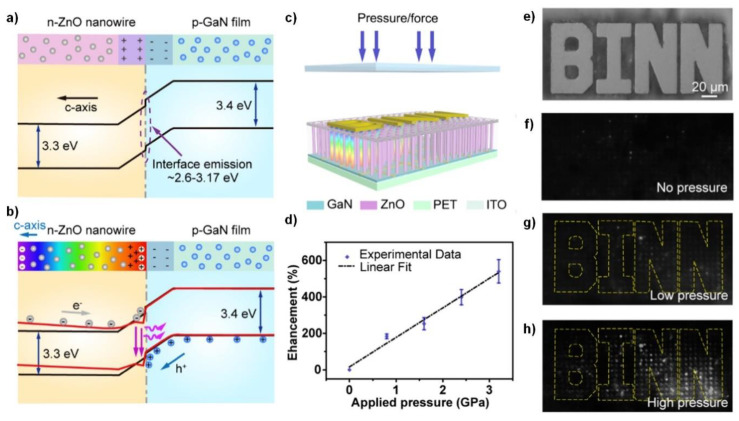
Piezophototronic-effect-assisted piezoelectric pressure imaging devices. (**a**,**b**) Energy band edge diagram of n-ZnO–p-GaN heterojunction before (**a**) and after (**b**) encountering compressive strain. (**c**) Device structure and strategy of selective pressure application. (**d**) Relationship between the enhancement factor E and the applied pressure. (**e**) Optical image of the sapphire seal used in measurements. (**f**–**h**) Electroluminescence images of the device at (**f**) zero, (**g**) low, and (**h**) high pressures, respectively. Reproduced from [38] with permission from Elsevier Limited. Copyright 2019.

A piezophototronic effect has been proposed as an innovative way of achieving high spatiotemporal resolution. Light emission from the p-n semiconductor heterojunctions based on GaN or InGaN has been widely used for solid-state lighting [35]. Because ZnO has a similar band-gap and as GaN, the n-ZnO–p-GaN heterojunction also exhibits light emitting diode (LED) characteristics when forward-biased [36]. If ZnO is subject to stress, the piezoelectric charge at the heterointerface introduces a local dip in the energy band edge at the junction. This distortion of the band tends to temporarily trap the holes and increases the carrier recombination rate, thus resulting in stronger light emission [37]. Furthermore, the piezoelectric charge works as an additional forward bias and increases the injection current. From the combinatory effect, the piezoelectric charge enhances the light emission, which indicates the amount of applied force. Figure 5a,b demonstrate this effect by schematic illustration of the energy band edge diagram of the n-ZnO–p-GaN heterojunction under idle (Figure 5a) and applied pressure (Figure 5b) situations [38]. Based on this effect, Peng et al. fabricated a flexible high-resolution pressure imaging device using a selectively grown dense ZnO NW array on a p-GaN thin film [38]. The entire device structure is illustrated in Figure 5c. Here, the p-GaN thin-film layer was prepared on a flexible poly(ethylene terephthalate) (PET) substrate by laser lift-off from a donor sapphire substrate. The hydrothermal method was employed to grow the ZnO NWs, with the c-axis pointing upwards. To complete the vertical device structure, the inter-nanowire space was filled with polymer (SU-8), and a transparent indium tin oxide (ITO) top common cathode was deposited at the top of the array. The Ni/Au grid bottom electrodes were deposited on the p-GaN for ohmic contact. The piezoelectric effect on light emission was quantified by defining an enhancement factor E as E = (I_p_-I_o_/I_o_), where I_o_ is defined as the light intensity of the LED without strain and I_p_ as the light intensity of the LED under applied stress. An approximately linear relationship between enhancement factor E and applied pressure was found, as shown in Figure 5d. To apply pressure in only the desired area, a 3D structured sapphire seal with an extruded pattern (Figure 5e) was used. Figure 5f–h show images demonstrating the operation of the piezophototronic device taken under zero, low, and high pressures, respectively, clearly indicating the ability to image the pressure distribution of the device. Recent research studies have employed p-type polymer or used CdS instead of ZnO, and enhancement of the light has been observed at relatively low pressures of 40–100 MPa [39,40,41]. Table 6 summarizes the pressure sensors based on the piezophototronic effect.

### 3.4. Summary

Piezoelectric nanowires paved the way for the development of miniaturized strain sensors to be organized as large-scale pressure sensor arrays and force imagers using simple Schottky diode structures. For strain sensing, dispersed nanowires with single or back-to-back Schottky junctions lead to outstanding sensitivity in arbitrary flexible substrates. However, scaling up such an approach from individual devices to arrays with large area coverage is challenging because current approaches require tracking the direction and morphology of every single nanowire. Systematic approaches might lead to statistically uniform performance, which is required for practical adoption of these devices, such as aligning the nanowires on textured substrates or integrating multiple nanowires into a single junction to average out the randomness of the dispersed nanowires. On the other hand, vertically aligned nanowire sensors rely on more scalable synthesis and fabrication techniques, which are appropriate for these applications. They can be fabricated into arrays of sensors, giving spatial information of the distribution of the applied force. Their performance depends on having a reliable 3D device structure, because vertically standing nanowires can be vulnerable to mechanical stress. Lastly, the formation of p-n junctions for piezoelectrically tunable LEDs provides innovative means to visualize external forces. They are free of electrical connections and can achieve superior spatiotemporal resolution. However, a high-performance image capturing unit such as a charge coupled device (CCD) is required to image the force and they have low sensitivity, which are issues that should be addressed for future applications, such as in signature pads and biometric security.

## 4. Thin-Film-Based Piezoelectric Devices

Thin-film-based devices have been widely employed in several industries, such as display, lighting, and radio frequency electronics [35,42,43]. Interestingly, materials such as ZnO and GaN, which are commonly used in thin-film devices, exhibit the piezoelectric effect. Accordingly, we foresee great potential for the commercialization of piezoelectric thin-film devices in the future, especially since they can be easily integrated with existing industrial processes. In addition, from the current achievement of thin-film-based electronics in display or mobile gadgets, we expect further advances in thin-film-based pressure and strain sensors, such as high-definition sensing on rigid or flexible substrates.

### 4.1. ZnO Thin-Film Transistors with Piezoelectric Sensing

ZnO has been widely employed in TFTs due to its excellent transparency and outstanding electronic properties. The main application of a variation of the ZnO TFT, the indium gallium zinc oxide (IGZO), is in displays, where TFTs control the individual pixels. At the same time, *c*-axis-aligned ZnO thin films can be grown using scalable methods, such as radio frequency (RF) magnetron sputtering or solution processes. Coupled with having a relatively high piezoelectric coefficient, this makes ZnO TFT an ideal candidate for electromechanical sensor applications.

#### 4.1.1. ZnO TFTs for Pressure Sensing

The application of ZnO TFTs as piezoelectric electromechanical sensors was demonstrated by Vishniakou et al. using standard microfabrication techniques, where the ZnO films were deposited by sputtering [44,45]. In this series of research, ZnO TFTs were structured into an active-matrix array. Because the channel of the TFT could be turned on and off by switching the gate bias, each TFT functioned as a sensor only when activated. This allowed the development of a pressure sensor array with a good refreshing rate and low crosstalk. The 8 × 8 active-matrix TFT array was fabricated on a glass substrate. This was connected to an 8 × 8 diaphragm actuator array, which reproduced the pressure input. From the readout of the TFT array data, the local maximum address was calculated and the corresponding actuator was deflected, which helped reproduce the touch input events. The reported on/off ratio of the device was 10^3^ and the on current level was 10^−7^ A. The signal-to-noise ratio (SNR) of 3 showed the sensitivity of the array to pressures extending to 15 kPa, a pressure which corresponds to the gentle human touch. This research showed the potential of a ZnO TFT array in digitizing the tactile input.

**Figure 6:**
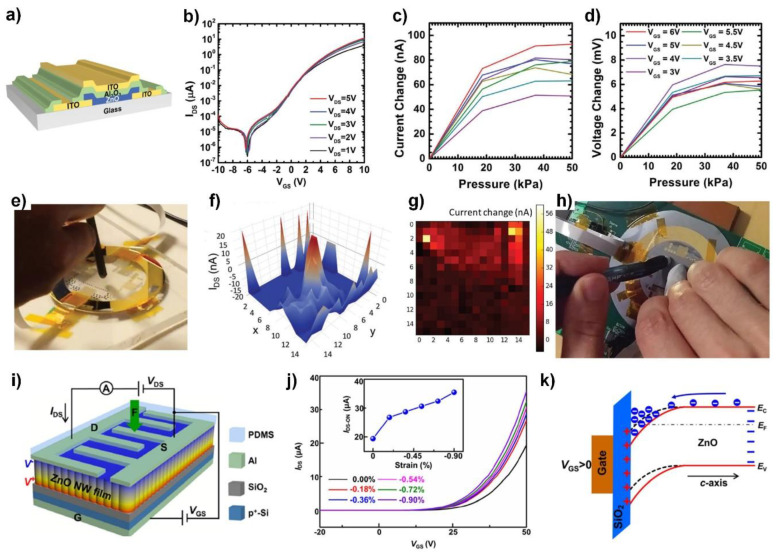
Piezoelectric current modulation in thin-film transistors in the work of Vishniakou et al. [45] (**a**–**h**) and Pan et al. [46] (**i**–**k**). (**a**) A schematic illustration of the device structure. (**b**) Transfer characteristic of the device. (**c**) Plot of current change as a function of pressure. (**d**) Calculated effective gate voltage as a function of pressure. (**e**) A photograph of local pressure being applied to the TFT array. (**f**) The 3D heatmap plot of current change in the TFT array. (**g**) Real-time heatmap of current change obtained by integrated circuit (IC) commercial driver. (**h**) Photograph of the device connected to the IC commercial driver. (**a**–**h**) Reproduced from [45] with permission from Wiley-VCH. Copyright 2018. (**i**) Schematic illustration of the bottom gate device. (**j**) Transfer curves as a function of strain. (**k**) Energy band edge diagram of the device, illustrating the accumulation of electrons near the bottom gate electrode. (**i**–**k**) Reproduced from [46] with permission from Elsevier Limited. Copyright 2018.

ZnO TFTs were further optimized for pressure sensing and the TFT fabrication processes were integrated with commercially available technologies, as introduced in Figure 6a–h. [45]. Here, the ZnO thin film was deposited by radio frequency (RF) magnetron sputtering with different ambient gas compositions and room temperature deposited seed layers. The optimized device exhibited high current modulation, with an *I_max_*/*I_min_* ratio of ≈ 10^5^ over a *V_GS_* range of 20 V, which is important for achieving low electrical crosstalk (Figure 6b). The sensitivity of the same device was approximately 4 nA kPa^−1^, with a latency of less than 1 ms (Figure 6c,d). A 16 × 16 active-matrix ZnO TFT array was fabricated on glass, as shown in Figure 6e. The heatmap of the current change from the array, generated by multiplexing the signal followed by image processing, represents the force distribution over the TFT array (Figure 6f). The array was further integrated with a commercial display driver chipset, which exhibited accurate spatial resolution (Figure 6g,h), with a refresh rate of ≈ 13 Hz. This demonstrated its potential as a new type of touch interface for interactive displays. Pan et al. (Figure 6i–k) [46] investigated the interplay between piezoelectric charges and the carrier concentration inside the channel. They observed that high carrier concentration suppresses the buildup of the piezoelectric potential due to charge screening effects. Alternatively, if the carrier concentration was too low, a negligible piezoelectric response was observed. These findings are consistent with the piezoelectric MIS TFT model in Section 2, where the high carrier density boosted the bulk conduction and diluted the effect of the $V_{FB}$ change. This study suggests that engineering the optimal carrier concentration is a key issue in the fabrication of the piezoelectric thin-film transistors.

#### 4.1.2. Applications in Robotics

**Figure 7:**
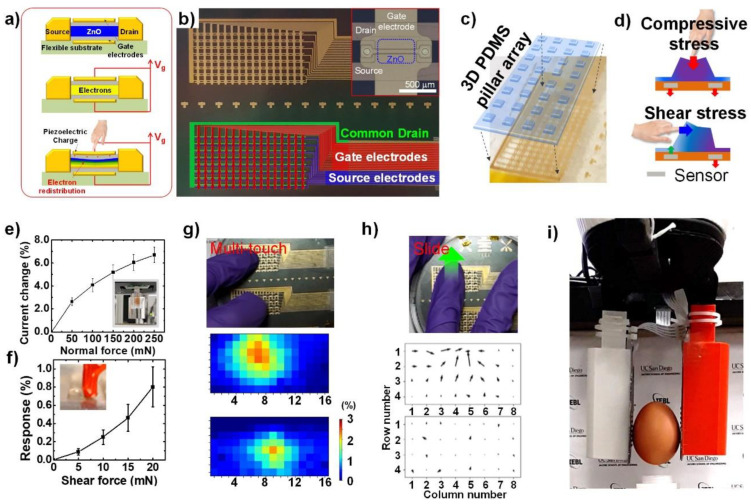
Pressure sensor array using an active-matrix field-effect transistor array with piezoelectric channels. (**a**) Cross-sectional schematic illustration of force sensing using dual-gated ZnO TFT. (**b**) Photograph of the fabricated active-matrix TFT array on 4-inch sized wafer. Inset is a magnified image of a single TFT. (**c**) Photograph of the 3D PDMS array placed on the TFT sensor array. (**d**) Strategy of measuring shear force applied on the TFT array using 3D PDMS bump array. (**e**) Average current change curve under different applied forces measured from 6 random elements in the array. (inset) A photograph of the experimental setup. (**f**) Average shear force vs. current change curve from six random unit cell. (inset) A close-up view of the tip applying in-plane force to the bump. (**g**) Response heatmaps of TFT arrays with double touch input. (**h**) Quiver plots of shear force when a finger slides over the TFT array. The direction of sliding finger is indicated as a green arrow. (**i**) Photograph of the robotic gripper holding a raw egg, which is equipped with the TFT array.

Recent progress on ZnO TFT pressure sensor arrays have utilized a dual-gate structure. Oh et al. developed two 8 × 16 dual-gate ZnO TFT array force sensors that recorded both the normal and shear force, and demonstrated a closed-loop control for robotic applications (Figure 7a,b) [47]. The dual-gated ZnO TFT provided better electrostatic control and sensing capability, as illustrated in Figure 7a. For shear-force sensing, a 3D Polydimethylsiloxane (PDMS) pillar array was deployed on every 2 × 2 sensor unit (Figure 7c). The applied shear force deformed the 3D PDMS pillar and generated a normal force gradient over the unit, which could be recalculated to estimate the applied shear force. The sensitivity for both normal (50–250 mN) and shear force (5–20 mN) was calculated and exhibited a linear behavior (Figure 7e,f). The device captured both the strength and direction of the haptic input (Figure 7g,h). More importantly, the device was integrated with a robotic gripper to provide normal and shear force data for feedback of the closed-loop control. The gripper was able to securely grasp fragile objects, such as a raw egg (Figure 7i), detect the slip at the interface, and adjust the grip force owing to its shear-force feedback ability.

### 4.2. GaN-Based Piezoelectric Transistors for Operation in Harsh Conditions

The GaN-based high electron mobility transistor (HEMT), or heterojunction field-effect transistor (HFET), is another important type of thin-film-based transistor. Historically, AlGaN/GaN HEMTs have been widely employed in RF communication, owing to their high electron mobility of two-dimensional electron gas (2DEG) located at the AlGaN/GaN heterointerface [48]. On the other hand, GaN, with its wurtzite structure similar to ZnO, is an excellent piezoelectric material. Therefore, external strain is expected to affect both the electron concentration and mobility of the 2DEG, thus altering its transfer properties. Because the 2DEG has high field-effect mobility, even incremental changes in 2DEG can result in a large change of the current.

Furthermore, the wide bandgap and excellent thermal stability of GaN allow its application in extreme conditions, such as under high temperatures (>200 °C), which is outside the range of normal operation for conventional Si devices [49]. Consequently, the GaN-based HEMT is a promising candidate for electromechanical sensors in harsh environments.

**Figure 8:**
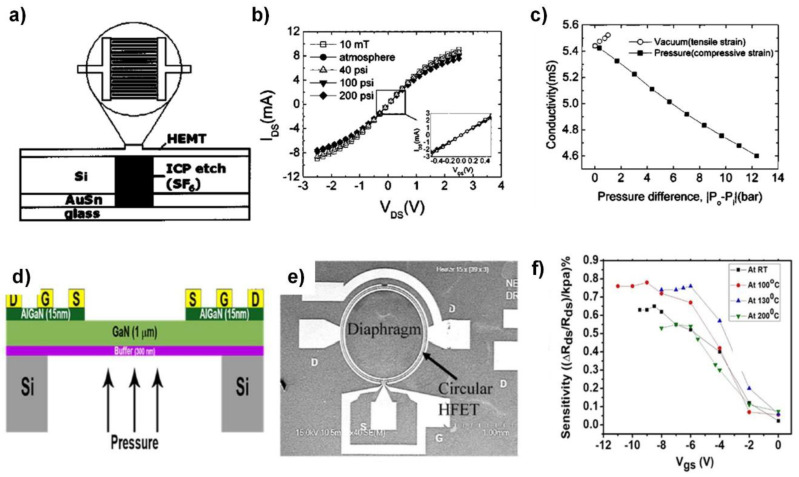
GaN-based devices for pressure sensing applications. (**a**) Cross-sectional diagram of the AlGaN/GaN HEMT device fabricated on a membrane on a Si substrate. (**b**) The I-V characteristics of the device as a function of applied pressure. (**c**) Channel conductivity of the HEMT membrane as a function of differential pressure. (**a**–**c**) Reproduced from [51] with permission from the American Institute of Physics. Copyright 2004. (**d**) Schematic illustration of ring-shaped AlGaN/GaN HFET. (**e**) SEM image in the channel area. (**f**) Sensitivity of the device measured at different temperatures. (**d**–**f**) Reproduced from [49] with permission from MDPI. Copyright 2018.

Investigations on force-induced current modulation in conventional AlGaN/GaN HEMT began in 2003 with Kang et al. [50]. In general, the current in 2DEG decreases under compressive strain and increases under tensile strain, due to the modulated energy band edge structure at the AlGaN/GaN heterojunction. For a maximal response, researchers developed a strategy of mounting the HEMT on a free-standing membrane [51]. The GaN-on-Si technology is suitable for this application, owing to the wide range of etch compatibility for Si. As shown in Figure 8a, the device exhibited a current change of ~ 10% when external pressure was applied (Figure 8b). The pressure vs. conductivity curve in Figure 8c clearly indicates the relation between the current and the applied pressure. Utilizing the robust physical properties of a GaN, recent work based on the same structure has demonstrated pressure sensing at 200 °C. Gajula et al. fabricated a circular AlGaN/GaN heterostructure field effect transistor on a freestanding diaphragm, as illustrated in Figure 8d,e [49]. The device exhibited excellent pressure sensing capability under high temperatures up to 200 °C. The sensitivity plot as a function of gate bias $V_{gs}$ shown in Figure 8f suggests that the device could transduce the pressure into the current change, and the sensitivity was tunable using the gate bias. Various pressure and strain sensors using a GaN-based HEMTs are summarized in Table 7.

### 4.3. Summary

ZnO-based TFTs and GaN thin-film-based HEMTs or HFETs are good candidates for future piezoelectric sensors because of their mature technology. ZnO TFT technologies can be applied to glass or flexible substrates using the same manufacturing tools as the current display technology. Additionally, the inherent multiplexing capability of ZnO TFTs allows active sensing matrices with single TFTs (sensor and multiplexer), faster refresh rates, and high on/off ratio. However, considering the brittle nature of oxides, application in stretchable electronics for use in wearable and biomedical applications might be challenging. GaN-based HEMTs or HFETs are unique piezoelectric sensor candidates for operation under harsh conditions. Despite the higher cost of their manufacture and the relatively low sensitivity, the technology is still valuable for specialized applications for operation under harsh conditions, such as in automobile, aerospace, and mining applications. Further studies would increase the progress of GaN-based piezoelectric devices for these applications.

## 5. 2D Materials and Ultrathin Nanofilms

The 2D materials have been extensively investigated in the last decade due to their unique crystal structures and physical properties. The asymmetric structures of a variety of binary 2D monolayers suggest a strong in-plane piezoelectricity. Furthermore, excellent physical properties of 2D monolayers, such as strong mechanical strength, tunable carrier dynamics, and excellent thermal and chemical stability, bring a synergetic effect to their piezoelectric device applications. At the same time, conventional materials can be thinned down into a few atomic layers. This is especially advantageous in wearable electronics, because ultrathin nanofilms can be easily integrated with flexible or stretchable substrates, without cracking or damaging the films.

### 5.1. Piezoelectricity in 2D TMDC

#### 5.1.1. Piezoelectric Coefficient of 2D TMDC

Transition metal dichalcogenides (TMDC) are a group of 2D materials known for their semiconducting electrical properties. In terms of the crystal structure, these are binary alloys formed in a hexagonal structure that can lack a center of symmetry along the in-plane direction. Accordingly, strong piezoelectricity appears in a single layer in TMDCs along the in-plane direction. However, no piezoelectricity is observed in the bulk form of these materials due to their stacking order. The piezoelectric dipoles of neighboring layers cancel each other.

The piezoelectric coefficient tensor of monolayer TMDCs was examined theoretically by Duerloo et al. [15,57]:

(24) $$d_{ij}=\left[\begin{array}{cccccc} d_{11} & -d_{11} & 0 & 0 & 0 & 0\\ 0 & 0 & 0 & 0 & 0 & -2d_{11}\\ 0 & 0 & 0 & 0 & 0 & 0 \end{array}\right]$$

The tensor equation indicates that in TMDCs, piezoelectric charges will be generated along the in-plane direction when an in-plane strain exists. Unlike conventional piezoelectric materials, in which $d_{33}$ dictates the piezoelectric response, here $d_{11}$ is the most relevant coefficient.

#### 5.1.2. TMDC Based Strain Sensors

**Figure 9:**
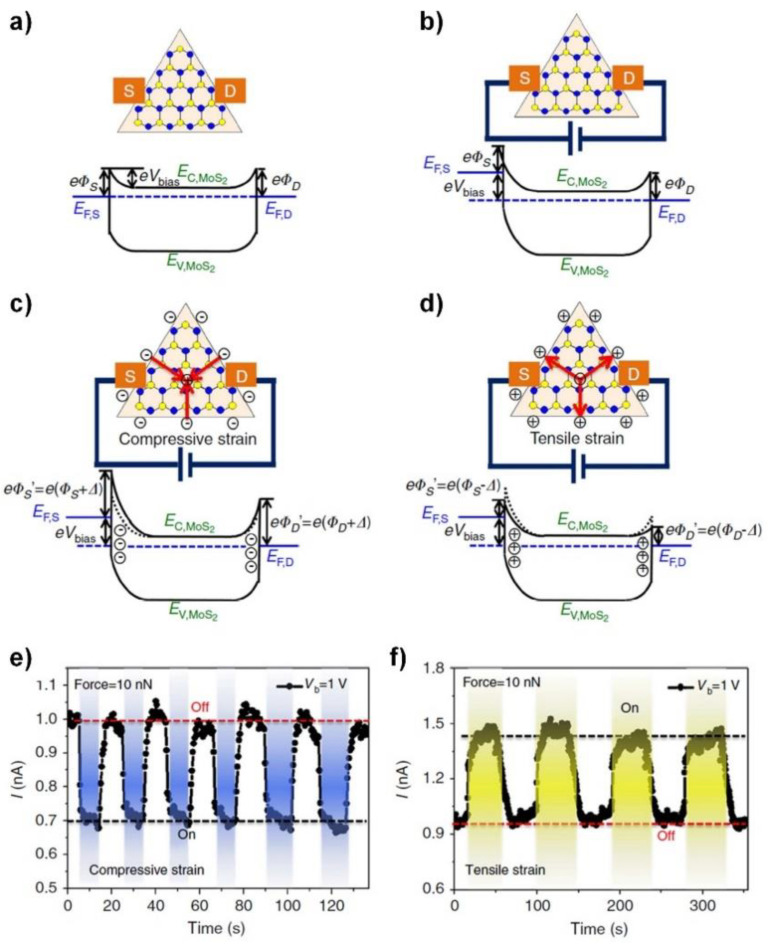
Transition metal dichalcogenide (TMDC) monolayer for piezoelectric device applications. (**a**–**d**) Energy band edge diagrams of the MoS_2_-monolayer-based piezoelectric device: (**a**) without bias voltage; (**b**) with external bias to the drain electrode; (**c**) with compressive strain applied to the monolayer; (**d**) with tensile strain applied to the monolayer. The red arrows represent the polarization direction. $E_{F}$, $E_{C}$, $E_{V}$, $V_{bias}$, and Δ represent the Fermi level, conduction band, valence band, external bias, and introduced piezopotential, respectively. Reproduced from [59] with permission from Springer Nature Limited. Copyright 2015.

A piezoelectric device made out of a single layer of TMDC was demonstrated using mechanically exfoliated MoS_2_ [58] and chemical vapor-deposited (CVD) MoS_2_ [59]. Qi et al. explained the strain sensing mechanism of CVD MoS_2_ monolayers using the schematic illustrations in Figure 9a–d [59]. Here, the source and drain Schottky contacts were placed at the zig-zag edges of the MoS_2_ (Figure 9a). Figure 9b shows the energy band edges with bias. If compressive strain is applied, negative charges will accumulate in the MoS_2_ at each contact and the SBH will increase, limiting the current across the barriers (Figure 9c). Conversely, tensile stress will reduce the SBH and increased current will flow across the barriers (Figure 9d). Figure 9e,f shows the temporal response of the device under multiple compressive and tensile stress cycles, where stresses were introduced by a force from the tip of an atomic force microscopy (AFM). The location of the tip determined the direction of the stress to the monolayer. As expected, an immediate decrease or increase of the current was observed when the loading force introduced compressive or tensile stress. Especially, a high gauge factor of ~1160 was achieved from the chemical vapor deposited (CVD) monolayer MoS_2_ strain sensors, suggesting the potential of 2D-material-based electromechanical sensors.

### 5.2. Flexoelectricity for Out-of-Plane Piezoelectric Effect

Out-of-plane electromechanical coupling is not expected in 2D materials due to their centrosymmetric structure along the c-axis direction. However, the polarization can still be generated along the surface normal due to the flexoelectric effect in the presence of a strain gradient. This is different from the piezoelectric effect, where a uniform strain generates the polarization. It has been difficult to investigate the flexoelectric effect in macroscale materials, as the required strain gradient is too large and can damage the material [57]. In 2D materials, on the other hand, even a small stress can generate a large strain gradient that can give rise to a measurable flexoelectric effect without breaking the material. Hence, it can be used as a new method of utilizing the out-of-plane electromechanical coupling in 2D materials in the absence of the $d_{33}$ coefficient.

**Figure 10:**
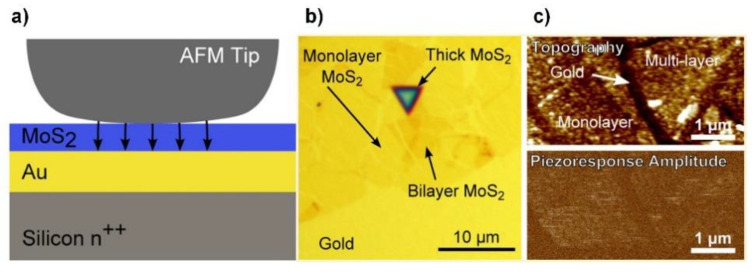
Flexoelectricity of the MoS_2_. (**a**) Schematic illustration of the measurement. Black arrows represent the electric field from the AFM tip. (**b**) Microscope image of MoS_2_ layers on gold film. (**c**) (**top**) Topography and (**bottom**) piezoresponse amplitude of the MoS_2_ layers on gold. Reproduced from [57] with permission from the American Chemical Society. Copyright 2017.

The flexoelectric effect in TMDCs was experimentally demonstrated by Brennan et al. using MoS_2_ [57]. The authors first experimentally measured the piezoelectric coefficient along the c-axis and then deduced the flexoelectric coefficient of the MoS_2_. In this experiment, piezoresponse force microscopy (PFM) was used. As shown in Figure 10a, the conductive AFM probe scanned the MoS_2_ layer, which was prepared on a gold-coated Si substrate, as shown in Figure 10b. To apply the electric field to MoS_2_, an oscillating drive voltage, $V_{d}$, was applied between the conductive probe of the AFM and the substrate. Figure 10c shows the topography of the MoS_2_ layers on gold (top) and the amount of deflection by the piezoelectric response (bottom). The deflection due to the applied electric field yielded a converse piezoelectric coefficient $d_{33}^{eff}$ of 1.0 ± 0.22 pm/V.

Since there is no d_33_ component in the MoS_2_ piezoelectric coefficient tensor, this response should be understood as a result of the flexoelectric effect. Mathematically, the flexoelectric effect can be explained using a fourth-rank tensor, compared to the conventional piezoelectric effect represented by a third-rank tensor [60]. The fourth-rank converse flexoelectric tensor for MoS_2_ is given by [61]

(25) $$\mu_{mn}^{*}=[\begin{array}{ccccccccc} \mu_{11}^{*} & 0 & 0 & 0 & \mu_{15}^{*} & 0 & 0 & 0 & \mu_{19}^{*}\\ \mu_{11}^{*} & 0 & 0 & 0 & \mu_{11}^{*} & 0 & 0 & 0 & \mu_{19}^{*}\\ \mu_{31}^{*} & 0 & 0 & 0 & \mu_{31}^{*} & 0 & 0 & 0 & \mu_{39}^{*}\\ 0 & 0 & 0 & 0 & 0 & \mu_{46}^{*} & 0 & \mu_{48}^{*} & 0\\ 0 & 0 & \mu_{46}^{*} & 0 & 0 & 0 & \mu_{48}^{*} & 0 & 0\\ 0 & \mu_{11}^{*}-\mu_{15}^{*} & 0 & \mu_{11}^{*}-\mu_{15}^{*} & 0 & 0 & 0 & 0 & 0 \end{array}]$$

Here, the * indicates that the converse representation is being used, and the indices are defined using the converse flexoelectric equation

(26) $$\sigma_{ij}=\mu_{ijkl}^{*}\frac{\partial E_{k}}{\partial x_{l}}$$

where $\sigma_{ij}$ is the stress tensor. The four indices can be transformed into two indices by using Voigt notation for *ij*, whereas *kl* follows 11→1, 12→2, 13→3, 21→4, 22→5, 23→6, 31→7, 32→8, 33→9 to yield $\mu_{mn}^{*}$ [57]. For a good approximation, a superposition of $\mu_{39}^{*}$ and $\mu_{48}^{*}$ contributes to the effective out-of-plane piezoelectric effect $d_{33}^{eff}$ value. For simplicity, the authors assumed that the response is mostly governed by $\mu_{39}^{*}$ and that it can be expressed as the effective flexoelectric coefficient $\mu_{eff}^{*}$. The following equation was used to calculate the $\mu_{eff}^{*}$.

(27) $$\mu_{eff}^{*}=d_{33}^{eff}Y\frac{t}{2}\;$$

where Y is the Young’s modulus and t is the thickness of the MoS_2_. From the measured $d_{33}^{eff}$ value, $\mu_{eff}^{*}$ was calculated to be 0.10 nC/m. This study confirmed the flexoelectricity of 2D materials in a quantitative way. This approach can be expanded to guide the design of strain sensors based on 2D materials, which can sense out-of-plane stress with tunable sensitivity.

### 5.3. Ultrathin ZnO Nanosheet Piezoelectrics

**Figure 11:**
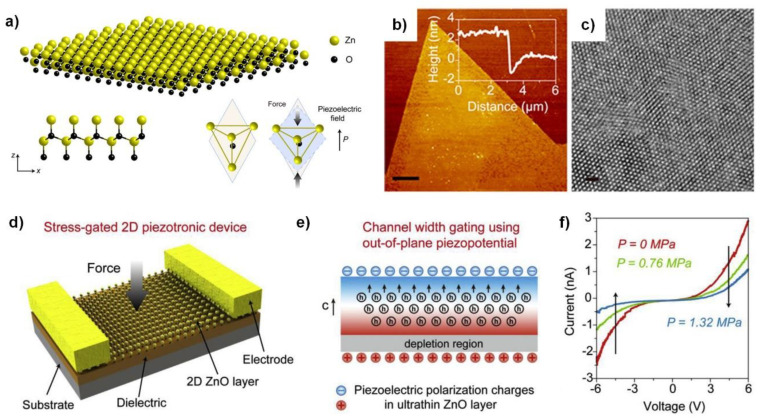
Atomically thin ZnO nanosheets for piezoelectric sensor applications. (**a**) Ultrathin ZnO materials with wurtzite structure and generation of a piezoelectric field under compressive pressure along the c-axis of the film. (**b**) AFM topography (Scale bar: 3 μm) and (**c**) high-resolution (HR) TEM image (Scale bar: 1 nm) of the ZnO ultrathin film. (**a**–**c**) Reproduced from [64] with permission from the American Chemical Society. Copyright 2018. (**d**) Device structure of the ultrathin ZnO-based device with channel width gating effect. (**e**) Schematic illustration of hole redistribution under compressive stress and the formation of the depletion region in ZnO ultrathin film. (f) I-V characteristics of the device under different applied pressure values. (**d**–**f**) Reproduced from [63] with permission from Elsevier Limited. Copyright 2019.

Recent advances in material synthesis have led to atomically thin ZnO nanosheets being achieved. Wang et al. reported that nanometer-thick ZnO film was synthesized via a solution-based technique, referred to as the water–air method [62]. The synthesis process was guided by surfactant monolayers at the water–air interface. The atomic configuration of this ultrathin ZnO nanosheet is shown in Figure 11a. The resulting film exhibited a thickness of 1–2 nm with a single crystalline structure, confirmed by the AFM measurement shown in Figure 11b and the high-resolution (HR) TEM study in Figure 11c. It should be noted that electrically the film was a p-type semiconductor, differing from bulk ZnO, which is usually n-type due to unwanted H-terminated oxygen vacancies. Except for its carrier type, the nanosheet maintains a wurtzite structure that is similar to its bulk form; thus, a piezoelectric response is expected when external strain is applied. Using this ultrathin nanosheet, piezoelectric devices with lateral channels were demonstrated [63]. The piezoelectric response was measured by applying force to the ZnO nanosheets, as shown in Figure 11d. Supported with finite element analysis, the positive piezoelectric charge depletes the nearby holes, while the accumulation effect of the negative piezoelectric charges is limited by screening of mobile holes (Figure 11e). The I-V curves with different applied pressure shown in Figure 11f confirm that the applied pressure reduces the current. Importantly, the piezoelectric effects were greatly enhanced in ZnO nanosheets, thus achieving a gauge factor of ~2 × 10^8^.

### 5.4. Summary

Due to their single atomic layer thickness affording extreme flexibility and due to the strong in-plane bonding, 2D materials are the ultimate choice for flexible or stretchable electromechanical sensors. Scalable synthesis of 2D materials, as well as precise control of their microstructure, such as the number of layers, orientation, and size of grains, are urgently required for practical applications. On the other hand, atomically thin ZnO is beneficial as it can encompass the advantages of both bulk and 2D materials. Uniform c-axis alignment allows for easy utilization of out-of-plane force for piezoelectricity, while its thinness means it is easily applied in flexible or stretchable electronics. The fundamental aspects of the mechanical and electrical properties need further investigations.

## 6. Summary and Outlook

Active piezoelectric devices have opened an avenue for markedly improved performance and additional functionalities in electromechanical sensors. Table 8 summarizes the advantages, disadvantages, challenges, operation range, and applications for different types of piezoelectric materials. The use of piezoelectric nanowires, such as ZnO nanowires, has generated an array of interesting applications, such as single-strain sensors, strain-operated logic gates, and high-density pressure sensor arrays. Integration with photonic devices led to piezophototronic devices, which can achieve high spatiotemporal resolution. Alternatively, thin-film-based devices armed with scalable vacuum technologies, such as TFT or HEMT processes, have acquired strain-sensing capability, thus bringing them closer to commercialization. Recently popularized ultrathin nanomaterials, such as monolayer 2D TMDC or ultrathin ZnO nanosheets, are gaining increased interest owing to their advantages from their unique physical properties. Additional opportunities for building unconventional, multifunctional devices are expected as 2D material research becomes more mature.

Nonetheless, many challenges must be solved for this technology to be used in practical applications. For nanowires or atom-thickness nanosheets, reliable material synthesis and device fabrication processes that can meet industrial standards are urgently required, despite their excellent physical properties and advancements. For thin-film-based devices, knowledge of the physics of strain sensing mechanisms is incomplete for device configurations, and so requires further investigation to achieve control over the sensitivity in various applications. Nevertheless, we expect a bright future for the development of active piezoelectric devices, as researchers and technologists seemingly will eventually improve their performance to meet the requirements for electromechanical sensors. Emerging industrial applications, such as healthcare devices, mobile and wearable electronics, and robotics for industrial and medical applications, will demand a variety of advanced electromechanical sensors. Simultaneously achieving high performance and a miniature size will be a key prerequisite for these applications.

In conclusion, strong advances have been achieved in active piezoelectric devices for electromechanical sensors. Moreover, relevant research continues to grow in terms of materials, device structures, and applications. Despite the remaining challenges, we believe that the technology will open a way to achieving the markedly improved electromechanical sensors required by the industry in the future.

## Figures and Tables

**Table 1:** Material dimensions of active piezoelectric devices.

Materials	Properties and Research Highlights
Nanowires	High aspect ratio, with length ranging between 0.1 and 100 μm. Single crystalline structure with asymmetry in axial direction. Piezoelectric charges modulate the fixed and free charge carriers at electrical contacts. Dispersed nanowires in vertically aligned nanowire array. Piezophototronic effect to achieve high spatiotemporal resolution.
Thin films	Uniform thickness, texture, and strong c-axis alignments over long range. Piezoelectric charge is induced on opposite surfaces of the film. Active devices, such as thin-film transistors (TFT), using zinc oxide (ZnO) or high-electron mobility transistors (HEMT) using gallium nitride (GaN).
2D nanosheets	Atomically thin (ranging from several angstrom to several nanometers) film, generally with an in-plane single crystalline structure. Piezoelectric charges are generated on specific atomic facets upon strain. Out-of-plane response induces flexoelectricity.

**Table 2:** Charge density, electric field, and potential of the metal–semiconductor Schottky junction with piezoelectric charges.

Position	Charge Density (ρ)	Electric Field (ε)	Electric Potential (Φ)
$0<x\leq \delta_{s}$	$\frac{\sigma^{PZ}}{\delta_{s}}+qN_{D}$	$\frac{1}{\varepsilon_{s}}\left[\begin{array}{c} \left(\frac{\sigma^{PZ}}{\delta_{s}}+qN_{D}\right)x\\ -qN_{D}W_{D}-\sigma^{PZ} \end{array}\right]$	$\frac{1}{\varepsilon_{s}}\left[\begin{array}{c} -\frac{1}{2}\left(\frac{\sigma^{PZ}}{\delta_{s}}+qN_{D}\right)x^{2}\\ +\left(qN_{D}W_{D}+\sigma^{PZ}\right)x \end{array}\right]$
$\delta_{s}<x\leq W_{D}$	$qN_{D}$	$\frac{q}{\varepsilon_{s}}N_{D}\left(x-W_{D}\right)$	$-\frac{q}{2\varepsilon_{s}}N_{D}{\left(x-W_{D}\right)}^{2}+\frac{\sigma^{PZ}\delta_{s}+qN_{D}W_{D}^{2}}{2\varepsilon_{s}}$
$x>W_{D}$	0	0	$\frac{\sigma^{PZ}\delta_{s}+qN_{D}W_{D}^{2}}{2\varepsilon_{s}}\;=V_{bi}-V$

**Table 3:** Charge density, electric field, and potential of the metal–insulator–semiconductor (MIS) structure with finite semiconductor thickness and piezoelectric charges.

Position	Charge Density (ρ)	Electric Field (ε)	Electric Potential (Φ)
−d<x<0	0	$-\frac{1}{\varepsilon_{i}}\left(qN_{D}W_{D}+\sigma^{PZ}\right)$	$\frac{1}{\varepsilon_{i}}\left(qN_{D}W_{D}+\sigma^{PZ}\right)\left(x+d\right)$
$0<x\leq \delta_{s}$	$\frac{\sigma^{PZ}}{\delta_{s}}+qN_{D}$	$-\frac{1}{\varepsilon_{s}}\left(qN_{D}W_{D}+\sigma^{PZ}\right)$ $+\frac{1}{\varepsilon_{s}}\left(\frac{\sigma^{PZ}}{\delta_{s}}+qN_{D}\right)x$	$\frac{1}{\varepsilon_{i}}\left(qN_{D}W_{D}+\sigma^{PZ}\right)d$ $+\frac{1}{\varepsilon_{s}}\left(qN_{D}W_{D}+\sigma^{PZ}\right)x$ $-\frac{1}{\varepsilon_{s}}\left(qN_{D}+\frac{\sigma^{PZ}}{\delta_{s}}\right)\frac{x^{2}}{2}$
$\delta_{s}<x\leq W_{D}$	$qN_{D}$	$\;\frac{1}{\varepsilon_{s}}qN_{D}\left(x-W_{D}\right)$	$\frac{1}{\varepsilon_{i}}\left(qN_{D}W_{D}+\sigma^{PZ}\right)d+\frac{1}{\varepsilon_{s}}\left(qN_{D}W_{D}+\sigma^{PZ}\right)x$ $-\frac{1}{\varepsilon_{s}}\sigma^{PZ}x-\frac{1}{\varepsilon_{s}}qN_{D}\frac{x^{2}}{2}+\frac{1}{2\varepsilon_{s}}\sigma^{PZ}\delta_{s}$
$W_{D}<x\leq t_{s}-W_{D}^{\prime }$	0	0	$\frac{1}{\varepsilon_{i}}\left(qN_{D}W_{D}+\sigma^{PZ}\right)d+\frac{qN_{D}W_{D}^{2}}{2\varepsilon_{s}}+\frac{1}{2\varepsilon_{s}}\sigma^{PZ}\delta_{s}$
$t_{s}-W_{D}^{\prime }<x\leq t_{s}-\delta_{s}$	$qN_{D}$	$\frac{1}{\varepsilon_{s}}qN_{D}\left(x-t_{s}+W_{D}^{\prime }\right)$	$\frac{1}{\varepsilon_{i}}\left(qN_{D}W_{D}+\sigma^{PZ}\right)d+\frac{qN_{D}W_{D}^{2}}{2\varepsilon_{s}}+\frac{1}{2\varepsilon_{s}}\sigma^{PZ}\delta_{s}$ $-\frac{1}{2\varepsilon_{s}}qN_{D}{\left(x-t_{s}+W_{D}^{\prime }\right)}^{2}$
$t_{s}-\delta_{s}<x\leq t_{s}$	$qN_{D}-\frac{{\sigma^{\prime }}^{PZ}}{\delta_{s}}$	$\frac{1}{\varepsilon_{s}}qN_{D}\left(W_{D}^{\prime }-\delta_{s}\right)+$ $\frac{1}{\varepsilon_{s}}\left(qN_{D}-\frac{\sigma \prime^{PZ}}{\delta_{s}}\right)$ $\times \left(x-t_{s}+\delta_{s}\right)$	$\frac{1}{\varepsilon_{i}}\left(qN_{D}W_{D}+\sigma^{PZ}\right)d+\frac{qN_{D}W_{D}^{2}}{2\varepsilon_{s}}+\frac{1}{2\varepsilon_{s}}\sigma^{PZ}\delta_{s}$ $-\frac{1}{2\varepsilon_{s}}qN_{D}{\left(W_{D}^{\prime }-\delta_{s}\right)}^{2}$ $-\frac{1}{\varepsilon_{s}}qN_{D}\left(W_{D}^{\prime }-\delta_{s}\right)\left(x-t_{s}+\delta_{s}\right)$ $-\frac{1}{2\varepsilon_{s}}\left(qN_{D}-\frac{{\sigma^{\prime }}^{PZ}}{\delta_{s}}\right){\left(x-t_{s}+\delta_{s}\right)}^{2}$

**Table 4:** Equations for $I_{DS}$ with different $V_{G}$ and $V_{D}$.

Range of $V_{G}$	Range of $V_{D}$	$I_{DS}$
$V_{T}<V_{G}\leq V_{FB}$	$V_{D}<V_{D,sat1}$	$I_{DS}=\frac{qN_{D}Z}{L}\mu_{b}\left\{\left(t_{s}+\frac{\varepsilon_{s}}{C_{i}}-\sqrt{\frac{2\varepsilon_{s}K}{qN_{D}}}\;\right)V_{D}-\frac{1}{4}\sqrt{\frac{2\varepsilon_{s}}{qN_{D}K}}V_{D}^{2}\right\}$
	$V_{D}\geq V_{D,sat1}$	$I_{DS}=\frac{qN_{D}Z}{L}\mu_{b}\sqrt{\frac{qN_{D}K}{2\varepsilon_{s}}}{\left(t_{s}+\frac{\varepsilon_{s}}{C_{i}}-\sqrt{\frac{2\varepsilon_{s}K}{qN_{D}}}\;\right)}^{2}$
$V_{G}>V_{FB}$	$V_{D}<V_{G}-V_{FB}$	$I_{DS}=\frac{Z}{L}\left\{\mu_{s}C_{i}\left(\left(V_{G}-V_{FB}\right)V_{D}-\frac{1}{2}V_{D}^{2}\right)+\mu_{b}qN_{D}t_{S}V_{D}\right\}$
	$V_{D}\geq V_{G}-V_{FB},$ $V_{D}<V_{D,sat2}$	$I_{DS}=\frac{Z}{L}\mu_{s}C_{i}\frac{{\left(V_{G}-V_{FB}\right)}^{2}}{2}$ $+\frac{qN_{D}Z}{L}\mu_{b}\left\{t_{s}V_{D}-\frac{C_{i}}{2qN_{D}}{\left(V_{D}-V_{G}+V_{FB}\right)}^{2}\right\}$
	$V_{D}\geq V_{D,sat2}$	$I_{DS}=\frac{Z}{L}\mu_{s}C_{i}\frac{{\left(V_{G}-V_{FB}\right)}^{2}}{2}+\frac{qN_{D}Z}{L}\mu_{b}t_{s}\left(V_{G}-V_{FB}+\frac{qN_{D}}{2C_{i}}t_{s}\right)$
$V_{T}=V_{FB}-\frac{qN_{D}}{2\varepsilon_{s}}\left[{\left(t_{s}+\frac{\varepsilon_{s}}{C_{i}}\right)}^{2}-{\left(\frac{\varepsilon_{s}}{C_{i}}\right)}^{2}\right]$, $K=V_{FB}-V_{G}+\frac{qN_{D}\varepsilon_{s}}{2C_{i}^{2}}$ $V_{D,sat1}=2\sqrt{\frac{qN_{D}}{2\varepsilon_{s}}}(t_{s}+\frac{\varepsilon_{s}}{C_{i}})-2K$, $V_{D,sat2}=V_{G}-V_{FB}+\frac{qN_{D}}{C_{i}}t_{s}$		

**Table 5:** Nanowire-based strain and pressure sensors.

Material	Device Type	Device Structure	Sensitivity	Etc.	References
ZnO NW	Strain sensor	Schottky back-to-back	Gauge factor 1250		[29]
ZnO NW	Strain sensor	Schottky back-to-back	~ 6 μA/strain change %	Logic gates	[30]
ZnO NW (Vertical)	Strain sensor	Schottky back-to-back, single Schottky			[32]
ZnO NW bundle array	Pressure sensor	Schottky contact passive-matrix array	2.1 μS kPa^−1^, Spatial resolution 100 μm	92 × 92 sensor array	[33]
ZnO NW bundle	Pressure sensor	MgO barrier layer(tunneling modulation)	7.1 × 10^4^ /9.81 mN Response time 128 ms		[34]

**Table 6:** Pressure sensors based on the piezophototronic effect.

Material	Sensitivity	Sensing Range	References
ZnO NW/p-GaN	12.88 GPa^−1^		[37]
ZnO NW/p-GaN	maximum 530%	0–3 GPa	[38]
ZnO NW/p-polymer		40–100 MPa	[39]
CdS NW/p-polymer	Enhancement factor increase ~20%/MPa (expected)	0–100 MPa	[41]
ZnO NW/OLED	Enhancement factor increase ~10%/MPa (expected)	0–100 MPa	[40]

**Table 7:** A comparison of GaN-based thin-film pressure and strain sensors.

Material Configuration	Device Type	Devise Structure	Sensitivity	Maximum Temperature	Reference
AlGaN/GaN	Strain sensing	HEMT on Cantilever	−6.0 ± 2.5 × 10^−10^ S/Pa (tensile) 9.5 ± 3.5 × 10^−10^ S/Pa (compressive)		[50]
AlGaN/GaN on Si	Pressure sensing	HEMT on membrane	−(+)6.4 × 10^−2^ mS/100kPa for compressive (tensile)		[51]
AlGaN/GaN	Pressure sensing	HFET	−8.0 mV/100MPa in threshold voltage		[52]
AlGaN/GaN	Pressure sensing	HEMT on diaphragm	I_d_ change of 38% at 5MPa		[53]
InAlN/GaN on Si	Pressure sensing	HEMT on diaphragm	0.64%/6.895 kPa		[54]
AlGaN/AlN/GaN Microwire	Strain sensing	Heterojunction Electron Gas device on cantilever	At room temperature: 165%/1.78% (compressive) 48%/1.78% (tensile)		[55]
AlGaN/GaN	Pressure sensing	HFET on diaphragm	0.022%/kPa (*V_g_* = 0 V) 0.76%/kPa (subthreshold region)	200 °C	[49]
GaN/AlGaN	Pressure	HEMT on diaphragm	0.02%/100kPa	55 °C	[56]

**Table 8:** Advantages, disadvantages, challenges, operation range, and applications for different types of piezoelectric materials.

Material Dimensionality	Device Type	Advantages, Disadvantages, and Challenges	Operation Range	Applications
Nanowires	ZnO NW Schottky diodes (Lateral)	**Advantages:** Simple 2-terminal device structure. Does not require high temperature after dispersion of NWs. Easy integration with glass or plastic substrate. **Disadvantages:** Dispersed NWs usually lack uniformity in direction, limits scalability. **Challenges:** Integration of uniformly dispersed NWs.	Strain ±~2%	Single strain/pressure sensors with high sensitivity
	ZnO NW Schottky diode array (Vertical)	**Advantages:** Solution based method yields NWs with uniform length and alignment over large area. Can form passive-matrix array. **Disadvantages:** Complicated process is required to fabricate 3D vertical structure. Vertical nanowires are vulnerable to large amounts of force **Challenges:** Simplifying the fabrication process for 3D structured device. Structural reinforcement to minimize mechanical degradation.	Pressure 0–50 kPa	Large scale pressure sensor array for haptic interfaces
	ZnO/GaN piezophototronics	**Advantages:** High spatial and temporal resolution can be achieved with relatively simple two-terminal structure. **Disadvantages:** Charge-coupled devices (CCDs) or other imaging devices are additionally required. Sensitivity is low (strong force is required) compared to other types of devices. **Challenges:** Improvement on sensitivity to kPa range.	(ZnO/GaN) Pressure 0–3 Gpa (Organic) 0–100 MPa	Signature pad, tablet, biomimetic security devices
Thin films	ZnO TFT	**Advantages:** Inherent multiplexing capability. Can construct active matrix structure without additional switching devices. **Disadvantages:** Complex process is required for fabrication of metal-insulator-semiconductor (MIS) transistor structure. **Challenges:** Further investigation on sensing mechanism.	Pressure 0–80 kPa	Large scale pressure sensor array with high spatiotemporal resolution for robotics, e-skin, haptic interfaces, etc.
	GaN HEMT	**Advantages:** Robust physical properties. **Disadvantages:** Poor sensitivity. High fabrication cost. **Challenges:** Device test under harsher condition is required.	Pressure 0–5 MPa	Strain/pressure sensor for harsh condition (Automobile, military, aerospace, etc.)
Ultrathin 2D materials	MoS_2_	**Advantages:** Simple 2-terminal devices. Suitable for flexible or stretchable devices. **Disadvantages:** Electrodes should be aligned at specific facets. Number of layers should be controlled (single layer is preferred). Interaction with out-of-plane force is complicated. **Challenges:** Synthesis of large area single-layer MoS_2_ with controlled crystal orientation can boost its electromechanical sensor applications.	Force 0–10 nN(by AFM) Strain ±~1% or less	Ultrathin sensors for extreme sensitivity (Microphone, fluid injection, etc.)
	ZnO ultrathin film	**Advantages:** Good for flexible or stretchable devices. Can easily utilize the out-of-plane force similar to thin films. **Disadvantages:** Material properties are not fully investigated. The synthesis method is unfamiliar yet. **Challenges:** Development of reliable scalable synthesis method. Fabrication of sensor array.	Pressure 0–1.3 MPa	

## Nomenclature

Symbol	Definition	SI Units
$A_{\;}$	The size of the area where external force is applied	m^2^
$A^{**}$	Effective Richardson constant	A/m^2^K^2^
$C_{i}$	Gate insulator capacitance per unit area	F/m^2^
$D_{s}$	Surface state density per unit area	states/ m^2^eV
$E_{g}$	Energy bandgap	eV
$F_{\;}$	Force	N
$G_{\;}$	Gauge factor	Dimensionless
$I_{DS}$	Current between source and drain	A
$I_{SF}$	Subthreshold current of the bulk	A
$I_{SR}$	Subthreshold current of the surface channel	A
$I_{b}$	Current in the bulk	A
$I_{s}$	Current in the surface channel	A
$J_{\;}$	Current density	A/m^2^
$J_{0}$	Saturation current density	A/m^2^
$J_{R}$	Current density in the reverse bias region	A/m^2^
$K_{\;}$	$V_{FB}-V_{G}+\frac{qN_{D}\varepsilon_{s}}{2C_{i}^{2}}$	V
$L_{\;}$	Length of the channel	m
$N_{D}$	Doping concentration of the semiconductor	/m^3^
$P_{i}$	Polarization field component	C/m^2^
$Q_{\;}$	Total free carriers in the film	C
$Q_{b}$	Free carriers in the bulk	C
$Q_{s}$	Free carriers in the accumulated channel	C
$Q_{t}$	Free carriers in the film without depletion region or accumulated channels	C
$T_{\;}$	Temperature	K
$V_{\;}$	Applied bias	V
$V_{D,sat1}$	Saturation voltage of the piezoelectric TFT when gate bias is below the flat-band voltage *V_FB_*	V
$V_{D,sat2}$	Saturation voltage of the piezoelectric TFT when gate bias is above the flat-band voltage $V_{FB}$	V
$V_{D}$	Drain bias	V
$V_{FB}$	Flat-band voltage	V
$V_{G}$	Gate bias	V
$V_{T}$	Threshold voltage	V
$V_{bi}$	Built-in potential between gate electrode and semiconductor	V
$W_{D}$	Depletion width at the metal–semiconductor interface (in Schottky diode) or at the gate insulator–semiconductor interface (in MIS TFT)	m
$W_{D}^{\prime }$	Depletion width of the semiconductor at the opposite side of the gate insulator–semiconductor interface (in MISTFT)	m
$Z_{\;}$	Width of the channel	m
$c_{2}$	A quantity containing interfacial properties	Dimensionless
$c_{ij}$	Coefficient of the stiffness tensor in Voigt notation	Pa
$d_{\;}$	Thickness of the gate insulator	m
$d_{ij}$	Piezoelectric moduli	C/N
$e_{ij}$	Piezoelectric tensor	C/m^2^
$h_{\;}$	Planck constant	J·s
ℏ	Reduced Planck constant (ℏ=h/2π)	J·s
$k_{\;}$	Boltzmann constant	m^2^kg/s^2^K
$n_{\;}$	Ideality factor	Dimensionless
$q_{\;}$	Elementary charge	C
$t_{s}$	Thickness of the semiconductor	m
$x_{\;}$	Relative position	m
$\delta_{i}$	Thickness of the interface trap charge	m
$\delta_{s}$	Thickness of the piezoelectric charge	m
$\varepsilon_{i}$	Permittivity of the gate insulator	F/m
$\varepsilon_{s}$	Permittivity of the semiconductor	F/m
$\mu_{b}$	Electron mobility of the bulk semiconductor	m^2^/Vs
$\mu_{s}$	Electron mobility of the accumulation channel	m^2^/Vs
$\sigma^{PZ}$	Piezoelectric charge density	C/m^2^
$\sigma_{i}$	Stress tensor	N/m^2^
$\varphi_{Bn}^{\prime }$	Schottky barrier height, including all effects	V
$\varphi_{Bn}^{0}$	Schottky barrier height without image force lowering or piezoelectric effect	V
$\chi_{i}$	Electron affinity of the insulator	V ($q\chi_{i}$: in eV)
$\chi_{s}$	Electron affinity of the semiconductor	V ($q\chi_{s}$: in eV)
$\psi_{s}$	Surface potential	V
$\epsilon_{i}$	Strain tensor component	Dimensionless
$\phi_{m}$	Work function of metal	V ($q\phi_{m}$: in eV)
$\phi_{s}$	Work function of semiconductor	V ($q\phi_{s}$: in eV)
$\Delta \varphi_{Bn}^{IFL}$	Change of Schottky barrier height due to image force lowering	V
$\Delta \varphi_{Bn}^{PZ}$	Change of Schottky barrier height due to piezoelectric charge	V
r	Reduction ratio of the back surface piezoelectric charge	Dimensionless

## Appendix A

### Appendix A1. Derivations of Charge Distribution and Related Properties in the Schottky Interface in Presence of Piezoelectric Charge

#### Appendix A1.1. Derivation of Charge Density, Electric Field, and Potential of the Metal–Semiconductor Schottky Junction with Piezoelectric Charges

In the presence of the piezoelectric charges, the charge density is given in Table A1, assuming that the piezoelectric charge is distributed over a distance, $\delta_{s}$, from the interface. Here, $W_{D}$ is the depletion width, while the position x is zero at the interface and positive inside the semiconductor.

**Table A1:** Charge density distribution of the piezoelectric Schottky interface.

Position	Charge Density (ρ)
$0<x\leq \delta_{s}$	$\frac{\sigma^{PZ}}{\delta_{s}}+qN_{D}$
$\delta_{s}<x\leq W_{D}$	$qN_{D}$
$W_{D}<x$	0

First, we start with Poisson’s equation:

(A1) $$\frac{d^{2}\Phi }{dx^{2}}=-\frac{\rho }{\varepsilon_{s}}$$

The electric field ε at position x can be derived by integration of the charge density:

(A2) $$\varepsilon \left(x\right)=-\frac{d\Phi }{dx}=-\int^{\;}\frac{d^{2}\Phi }{dx^{2}}dx=\int^{\;}\frac{\rho \left(x\right)}{\varepsilon_{s}}dx=\frac{\rho \left(x\right)}{\varepsilon_{s}}x+C$$

The constant *C* can be obtained from the boundary conditions, whereby ε should be continuous and ε=0 for W<x.

**Table A2:** Derivations of the electric field distribution of the piezoelectric Schottky interface.

Position	Indefinite Integral Form of Electric Field	Constants	Electric Field (ε)
$0<x\leq \delta_{s}$	$\frac{1}{\varepsilon_{s}}\left(\frac{\sigma^{PZ}}{\delta_{s}}+qN_{D}\right)x+C_{1}$	$C_{1}=-\frac{qN_{D}W_{D}+\sigma^{PZ}}{\varepsilon_{s}}$	$\frac{1}{\varepsilon_{s}}\left[\begin{array}{c} \left(\frac{\sigma^{PZ}}{q\delta_{s}}+qN_{D}\right)x\\ -qN_{D}W_{D}-\sigma^{PZ} \end{array}\right]$
$\delta_{s}<x\leq W_{D}$	$\frac{qN_{D}x}{\varepsilon_{s}}+C_{2}$	$C_{2}=-\frac{qN_{D}W_{D}}{\varepsilon_{s}}$	$\frac{q}{\varepsilon_{s}}N_{D}\left(x-W_{D}\right)$
$x>W_{D}$	0	0	0

In the same manner, the electric potential Φ can be obtained by integrating the electric field:

(A3) $$\Phi \left(x\right)=\int^{\;}\frac{d\Phi }{dx}dx=\int^{\;}-\varepsilon \left(x\right)dx$$

The boundary conditions should be applied, whereby Φ=0 for x≤0, $\Phi =V_{\mathrm{bi}}-V$ for x>0, and Φ should be continuous.

**Table A3:** Derivations of electric potential distribution of the piezoelectric Schottky interface.

Position	Indefinite Integral Form of Electric Potential	Constants	Electric Potential (Φ)
$0<x\leq \delta_{s}$	$\frac{1}{\varepsilon_{s}}\left[\begin{array}{c} -\frac{\left(\frac{\sigma^{PZ}}{\delta_{s}}+qN_{D}\right)}{2}x^{2}\\ +\left(qN_{D}W_{D}+\sigma^{PZ}\right)x\\ +C_{1} \end{array}\right]$	$C_{1}=0$	$\frac{1}{\varepsilon_{s}}\left[\begin{array}{c} -\frac{\left(\frac{\sigma^{PZ}}{\delta_{s}}+qN_{D}\right)}{2}x^{2}\\ +\left(qN_{D}W_{D}+\sigma^{PZ}\right)x \end{array}\right]$
$\delta_{s}<x\leq W_{D}$	$-\frac{q}{2\varepsilon_{s}}N_{D}{\left(x-W_{D}\right)}^{2}+C_{2}$	$C_{2}=\frac{\sigma^{PZ}\delta_{s}+qN_{D}W_{D}^{2}}{2\varepsilon_{s}}$	$-\frac{q}{2\varepsilon_{s}}N_{D}{\left(x-W_{D}\right)}^{2}+\frac{\sigma^{PZ}\delta_{s}+qN_{D}W_{D}^{2}}{2\varepsilon_{s}}$
$x>W_{D}$	$C_{3}$	$C_{3}=C_{2}=V_{bi}-V$	$\frac{\sigma^{PZ}\delta_{s}+qN_{D}W_{D}^{2}}{2\varepsilon_{s}}$ $=V_{bi}-V$

Combining the three tables gives the set of solutions provided in the Table 2.

### Appendix A1.2. Derivation of Depletion Width and Schottky Barrier Height in the Presence of the Piezoelectric Charge

The depletion width is derived from the equation:

(A4) $$\frac{\sigma^{PZ}\delta_{s}+qN_{D}W_{D}^{2}}{2\varepsilon_{s}}=V_{bi}-V$$

Solving this for $W_{D}$ gives:

(A5) $$W_{D}=\sqrt{\frac{2\varepsilon_{s}\left(V_{bi}-V\right)}{qN_{D}}-\frac{\sigma^{PZ}\delta_{s}}{qN_{D}}\;}$$

The shift of the SBH from to the piezoelectric charge is obtained by calculating the potential drop due to the piezoelectric charge. Since the total piezoelectric charge $\sigma^{PZ}$ is distributed over thickness of $\delta_{s}$, the potential drop can be expressed as:

(A6) $$\Delta \varphi_{Bn}^{PZ}=-\frac{1}{\varepsilon_{s}}\int_{0}^{\delta_{s}}\frac{\sigma^{PZ}}{\delta_{s}}xdx=-\frac{\sigma^{PZ}\delta_{s}}{2\varepsilon_{s}}$$

### Appendix A1.3. Parameters for Figure 1

The following are universal constants used to generate plots in Figure 1 and Figure 2.

**Table A4:** Universal constants used in the plots in Figure 1 and Figure 2.

Symbol	Parameter	Value	Notes
$q_{\;}$	Elementary charge	1.60 × 10^−19^ C	
$\varepsilon_{0}$	Vacuum permittivity	8.85 × 10^−12^ C/Vm	
$k_{\;}$	Boltzmann constant	1.38 × 10^−23^ m^2^ kg/s^2^K	
$m_{0}$	Electron mass	9.11 × 10^−31^ kg	

The following is the list of parameters used to generate plots in Figure 1.

**Table A5:** List of parameters used in plots of Figure 1.

Symbol	Parameter	Value	Notes
$N_{D}$	Doping concentration of the semiconductor	1 × 10^17^cm^−3^	Assumption
$q\phi_{m}$	Work function of metal	5.1 eV	
$q\chi_{s}$	Electron affinity of the semiconductor	4.3 eV	
$q\phi_{s}$	Work function of semiconductor	4.76 eV	Calculated from the carrier concentration, energy bandgap, effective electron and hole masses
$T_{\;}$	Temperature	300 K	
$\varepsilon_{s}$	Permittivity of the semiconductor	8.5$\varepsilon_{0}$	
$m_{e}$	Effective electron mass of the semiconductor	0.19$m_{0}$	
$m_{h}$	Effective hole mass of the semiconductor	0.21$m_{0}$	
$E_{g}$	Energy bandgap	3.35 eV	
$d_{33}$	Piezoelectric moduli in z-axis direction	12.4 pC/N	
F/A	Applied pressure to the junction	200 MPa	
$\delta_{s}$	Thickness of the piezoelectric charge	0.5 nm	Assumption

To calculate the work function of the semiconductor, $q\phi_{s},\;$the below equation was used [21], where the intrinsic carrier concentration $n_{i}$ of the semiconductor is

(A7) $$n_{i}=2{\left(\frac{kT}{2\pi \hbar^{2}}\right)}^{\frac{3}{2}}{\left(m_{e}m_{h}\right)}^{\frac{3}{4}}\exp\left(-\frac{E_{g}}{2kT}\right)\;$$

Semiconductors with an electron concentration of $N_{D}$ are as below:

(A8) $$E_{F}-E_{i}=kT\ln\left(\frac{N_{D}}{n_{i}}\right)\;$$

Finally, the work function of the semiconductor is:

(A9) $$q\phi_{s}=q\chi_{s}+\frac{1}{2}E_{g}-\left(E_{F}-E_{i}\right)$$

### Appendix A2. Derivations of Charge Distribution and Related Properties in the Piezoelectric Thin-Film Transistor

#### Appendix A2.1. Derivation of Charge Density, Electric Field, and Potential of the Metal–Semiconductor Schottky Junction with Piezoelectric Charges

In the presence of the piezoelectric charges, the charge density across the MIS structure is given in the following table, assuming that the piezoelectric charge is distributed over the length of $\delta_{s}$ from each surface. Here, $W_{D}$ and ${W_{D}}^{\prime }$ are the depletion widths of top and bottom surfaces of the channel, respectively. Position x is zero at the semiconductor–gate dielectric interface and positive on the semiconductor side.

**Table A6:** Charge density distribution of the piezoelectric MIS structure.

Position	Charge Density (ρ)
−d<x<0	0
$0<x\leq \delta_{s}$	$\frac{\sigma^{PZ}}{\delta_{s}}+qN_{D}$
$\delta_{s}<x\leq W_{D}$	$qN_{D}$
$W_{D}<x\leq t_{s}-{W_{D}}^{\prime }$	0
$t_{s}-W_{D}^{\prime }<x\leq t_{s}-\delta_{s}$	$qN_{D}$
$t_{s}-\delta_{s}<x\leq t_{s}$	$qN_{D}-\frac{{\sigma^{\prime }}^{PZ}}{\delta_{s}}$

The electric field and potential can be obtained in the same manner as equations are derived for the piezoelectric Schottky interface.

**Table A7:** Derivations of electric field distribution of the piezoelectric MIS structure.

Position	Indefinite Integral Form of Electric Field	Constants	Electric Field (ε)
−d<x<0	$C_{1}$	$C_{1}=-\frac{1}{\varepsilon_{i}}\left(qN_{D}W_{D}+\sigma^{PZ}\right)$	$-\frac{1}{\varepsilon_{i}}\left(qN_{D}W_{D}+\sigma^{PZ}\right)$
$0<x\leq \delta_{s}$	$\frac{1}{\varepsilon_{s}}\left(\frac{\sigma^{PZ}}{\delta_{s}}+qN_{D}\right)x+C_{2}$	$C_{2}=-\frac{1}{\varepsilon_{s}}\left(qN_{D}W_{D}+\sigma^{PZ}\right)$	$-\frac{1}{\varepsilon_{s}}\left(qN_{D}W_{D}+\sigma^{PZ}\right)$ $+\frac{1}{\varepsilon_{s}}\left(\frac{\sigma^{PZ}}{\delta_{s}}+qN_{D}\right)x$
$\delta_{s}<x\leq W_{D}$	$\frac{1}{\varepsilon_{s}}qN_{D}x+C_{3}$	$C_{3}=-\frac{1}{\varepsilon_{s}}qN_{D}W_{D}$	$\frac{1}{\varepsilon_{s}}qN_{D}\left(x-W_{D}\right)$
$W<x\leq t_{s}-{W_{D}}^{\prime }$	$C_{4}$	$C_{4}=0$	0
$t_{s}-W_{D}^{\prime }<x\leq t_{s}-\delta_{s}$	$\frac{1}{\varepsilon_{s}}qN_{D}x+C_{5}$	$C_{5}=-\frac{1}{\varepsilon_{s}}qN_{D}\left(t_{s}-W_{D}^{\prime }\right)$	$\frac{1}{\varepsilon_{s}}qN_{D}\left(x-t_{s}+W_{D}^{\prime }\right)$
$t_{s}-\delta_{s}<x\leq t_{s}$	$\frac{1}{\varepsilon_{s}}\left(qN_{D}-\frac{{\sigma^{\prime }}^{PZ}}{\delta_{s}}\right)x+C_{6}$	$C_{6}=\frac{1}{\varepsilon_{s}}qN_{D}\left(W_{D}^{\prime }-\delta_{s}\right)+$ $-\frac{1}{\varepsilon_{s}}\left(qN_{D}-\frac{{\sigma^{\prime }}^{PZ}}{\delta_{s}}\right)$ $\times \left(t_{s}-\delta_{s}\right)$	$\frac{1}{\varepsilon_{s}}qN_{D}\left(W_{D}^{\prime }-\delta_{s}\right)+$ $\frac{1}{\varepsilon_{s}}\left(qN_{D}-\frac{{\sigma^{\prime }}^{PZ}}{\delta_{s}}\right)$ $\times \left(x-t_{s}+\delta_{s}\right)$

It should be noted that at the dielectric-semiconductor interface (x=0), there is a discontinuity in the electric field due to discontinuous electric permittivity.

**Table A8:** Derivations of electric potential distribution of the piezoelectric MIS structure.

Position	Indefinite Integral Form of Electric Potential	Constants	Electric Potential (Φ)
−d<x<0	$\frac{1}{\varepsilon_{i}}\left(qN_{D}W_{D}+\sigma^{PZ}\right)x+C_{1}$	$C_{1}=\frac{1}{\varepsilon_{i}}\left(qN_{D}W_{D}+\sigma^{PZ}\right)d$	$\frac{1}{\varepsilon_{i}}\left(qN_{D}W_{D}+\sigma^{PZ}\right)\left(x+d\right)$
$0<x\leq \delta_{s}$	$\frac{1}{\varepsilon_{s}}\left(qN_{D}W_{D}+\sigma^{PZ}\right)x$ $-\frac{1}{\varepsilon_{s}}\left(\frac{\sigma^{PZ}}{\delta_{s}}+qN_{D}\right)\frac{x^{2}}{2}+C_{2}$	$C_{2}=C_{1}=$ $\frac{1}{\varepsilon_{i}}\left(qN_{D}W_{D}+\sigma^{PZ}\right)d$	$\frac{1}{\varepsilon_{i}}\left(qN_{D}W_{D}+\sigma^{PZ}\right)d$ $+\frac{1}{\varepsilon_{s}}\left(qN_{D}W_{D}+\sigma^{PZ}\right)x$ $-\frac{1}{\varepsilon_{s}}\left(qN_{D}+\frac{\sigma^{PZ}}{\delta_{s}}\right)\frac{x^{2}}{2}$
$\delta_{s}<x\leq W_{D}$	$-\frac{1}{\varepsilon_{s}}qN_{D}\frac{{\left(x-W_{D}\right)}^{2}}{2}+C_{3}\;$	$C_{3}=\frac{1}{\varepsilon_{i}}\left(qN_{D}W_{D}+\sigma^{PZ}\right)d$ $+\frac{qN_{D}W_{D}^{2}}{2\varepsilon_{s}}+\frac{1}{2\varepsilon_{s}}\sigma^{PZ}\delta_{s}$	$\frac{1}{\varepsilon_{i}}\left(qN_{D}W_{D}+\sigma^{PZ}\right)d$ $+\frac{1}{\varepsilon_{s}}qN_{D}W_{D}x-\frac{1}{\varepsilon_{s}}qN_{D}\frac{x^{2}}{2}$ $+\frac{1}{2\varepsilon_{s}}\sigma^{PZ}\delta_{s}$
$W_{D}<x\leq t_{s}-W_{D}^{\prime }$	$C_{4}$	$C_{4}=C_{3}$	$\frac{1}{\varepsilon_{i}}\left(qN_{D}W_{D}+\sigma^{PZ}\right)d$ $+\frac{qN_{D}W_{D}^{2}}{2\varepsilon_{s}}+\frac{1}{2\varepsilon_{s}}\sigma^{PZ}\delta_{s}$
$t_{s}-W_{D}^{\prime }<x\leq t_{s}-\delta_{s}$	$-\frac{1}{\varepsilon_{s}}qN_{D}\frac{{\left(x-t_{s}+W_{D}^{\prime }\right)}^{2}}{2}$ $+C_{5}$	$C_{5}=C_{3}$	$\frac{1}{\varepsilon_{i}}\left(qN_{D}W_{D}+\sigma^{PZ}\right)d$ $+\frac{qN_{D}W_{D}^{2}}{2\varepsilon_{s}}+\frac{1}{2\varepsilon_{s}}\sigma^{PZ}\delta_{s}$ $-\frac{1}{2\varepsilon_{s}}qN_{D}{\left(x-t_{s}+W_{D}^{\prime }\right)}^{2}$
$t_{s}-\delta_{s}<x\leq t_{s}$	$-\frac{1}{\varepsilon_{s}}qN_{D}\left(W_{D}^{\prime }-\delta_{s}\right)x$ $-\frac{1}{\varepsilon_{s}}\left(qN_{D}-\frac{{\sigma^{\prime }}^{PZ}}{\delta_{s}}\right)$ $\times \frac{{\left(x-t_{s}+\delta_{s}\right)}^{2}}{2}+C_{6}$	$C_{6}=C_{3}$ $-\frac{1}{2\varepsilon_{s}}qN_{D}{\left(W_{D}^{\prime }-\delta_{s}\right)}^{2}$ $-\frac{1}{\varepsilon_{s}}qN_{D}\left(W_{D}^{\prime }-\delta_{s}\right)$ $\times \left(-t_{s}+\delta_{s}\right)$	$\frac{1}{\varepsilon_{i}}\left(qN_{D}W_{D}+\sigma^{PZ}\right)d$ $+\frac{qN_{D}W_{D}^{2}}{2\varepsilon_{s}}+\frac{1}{2\varepsilon_{s}}\sigma^{PZ}\delta_{s}$ $-\frac{1}{2\varepsilon_{s}}qN_{D}{\left(W_{D}^{\prime }-\delta_{s}\right)}^{2}$ $-\frac{1}{\varepsilon_{s}}qN_{D}\left(W_{D}^{\prime }-\delta_{s}\right)$ $\times \left(x-t_{s}+\delta_{s}\right)$ $-\frac{1}{2\varepsilon_{s}}\left(qN_{D}-\frac{{\sigma^{\prime }}^{PZ}}{\delta_{s}}\right)$ $\times {\left(x-t_{s}+\delta_{s}\right)}^{2}$

### Appendix A2.2. Derivation of Current Equations in the Piezoelectric TFT

#### Appendix A2.2.1. $V_{T}<V_{G}<V_{FB}$ (Bulk Conduction Only)

First, $V_{T}$ is defined as the potential required for the whole thin film thickness to be depleted, i.e., $W_{D}=t_{s}$,

(A10) $$t_{s}=\sqrt{\frac{2\varepsilon_{s}}{qN_{D}}\left(V_{FB}-V_{T}\right)+{\left(\frac{\varepsilon_{s}}{C_{i}}\right)}^{2}}-\frac{\varepsilon_{s}}{C_{i}}\;V_{T}=V_{FB}-\frac{qN_{D}}{2\varepsilon_{s}}\left[{\left(t_{s}+\frac{\varepsilon_{s}}{C_{i}}\right)}^{2}-{\left(\frac{\varepsilon_{s}}{C_{i}}\right)}^{2}\right]=V_{FB}-\frac{qN_{D}}{2\varepsilon_{s}}t_{s}\left(t_{s}+2\frac{\varepsilon_{s}}{C_{i}}\right)$$

Let $Q_{b}$ be the amount of charge due to free carriers in the film. Here, $t_{s}$ is the thickness of the film and W_D_ is the depletion width. Since there is no surface channel, the amount of free carriers Q participating in the current is:

(A11) $$Q=Q_{b}=Q_{t}-Q_{D}$$

Here, $Q_{t}$ is the total amount of free carriers in the film when there is no depletion and $Q_{D}$ is the amount of charge in the depletion region:

(A12) $$Q_{b}=qN_{D}\left(t_{s}-W\left(V_{G},\Delta \psi \left(y\right)\right)\right)$$

Here, $W_{D}$ can be expressed as:

(A13) $$W_{D}\left(V_{G},\Delta \psi \left(i\right)\right)=\sqrt{\frac{2\varepsilon_{s}}{qN_{D}}\left(V_{FB}-V_{G}+\Delta \psi \left(y\right)\right)+{\left(\frac{\varepsilon_{s}}{C_{i}}\right)}^{2}}-\frac{\varepsilon_{s}}{C_{i}}$$

The current in this bulk conduction regime can be calculated using $\mu_{b}$ as the bulk mobility, as follows:

(A14) $$I_{DS}=\frac{qN_{D}Z}{L}\mu_{b}\int_{0}^{V_{D}}\left(t_{s}-W\left(V_{G},\Delta \psi \left(y\right)\right)\right)d\Delta \psi \left(y\right)\;=\frac{qN_{D}Z}{L}\mu_{b}\int_{0}^{V_{D}}\left(t_{s}+\frac{\varepsilon_{s}}{C_{i}}-\sqrt{\frac{2\varepsilon_{s}}{qN_{D}}\left(V_{FB}-V_{G}+\Delta \psi \left(y\right)\right)+{\left(\frac{\varepsilon_{s}}{C_{i}}\right)}^{2}}\right)d\Delta \psi \left(y\right)$$

Continuing the integration:

(A15) $$I_{DS}=\frac{qN_{D}Z}{L}\mu_{b}{\left[\left(t_{s}+\frac{\varepsilon_{s}}{C_{i}}\right)\Delta \psi \left(y\right)-\sqrt{\frac{2\varepsilon_{s}}{qN_{D}}}\frac{2}{3}\;{\left(V_{FB}-V_{G}+\Delta \psi \left(y\right)+\frac{qN_{D}}{2\varepsilon_{s}}{\left(\frac{\varepsilon_{s}}{C_{i}}\right)}^{2}\right)}^{\frac{3}{2}}\right]}_{0}^{V_{D}}\;$$

Let:

(A16) $$K\equiv V_{FB}-V_{G}+{\left(\frac{\varepsilon_{s}}{C_{i}}\right)}^{2}\frac{qN_{D}}{2\varepsilon_{s}}=V_{FB}-V_{G}+\frac{qN_{D}\varepsilon_{s}}{2C_{i}^{2}}$$

Then:

(A17) $$\begin{array}{ll} I_{DS} & =\frac{qN_{D}Z}{L}\mu_{b}{\left[\left(t_{s}+\frac{\varepsilon_{s}}{C_{i}}\right)\Delta \psi \left(y\right)-\frac{2}{3}\sqrt{\frac{2\varepsilon_{s}}{qN_{D}}}\;K^{\frac{3}{2}}\;{\left(1+\frac{\Delta \psi \left(y\right)}{K}\right)}^{\frac{3}{2}}\right]}_{0}^{V_{D}}\\ & =\frac{qN_{D}Z}{L}\mu_{b}\left\{\left(t_{s}+\frac{\varepsilon_{s}}{C_{i}}\right)V_{D}-\frac{2}{3}\sqrt{\frac{2\varepsilon_{s}}{qN_{D}}}\;K^{\frac{3}{2}}\left({\left(1+\frac{V_{D}}{K}\right)}^{\frac{3}{2}}-1\right)\right\} \end{array}$$

For small $\frac{V_{D}}{K}$, binomial approximation of the second order gives:

(A18) $$I_{DS}=\frac{qN_{D}Z}{L}\mu_{b}\left\{\left(t_{s}+\frac{\varepsilon_{s}}{C_{i}}\right)V_{D}-\frac{2}{3}\sqrt{\frac{2\varepsilon_{s}}{qN_{D}}}\;K^{\frac{3}{2}}\left(1+\frac{3}{2}\frac{V_{D}}{K}+\frac{1}{2}\frac{3}{2}\;\left(\frac{3}{2}-1\right){\left(\frac{V_{D}}{K}\right)}^{2}-1\right)\right\}\;$$

Finally:

(A19) $$I_{DS}=\frac{qN_{D}Z}{L}\mu_{b}\left\{\left(t_{s}+\frac{\varepsilon_{s}}{C_{i}}-\sqrt{\frac{2\varepsilon_{s}K}{qN_{D}}}\;\right)V_{D}-\frac{1}{4}\sqrt{\frac{2\varepsilon_{s}}{qN_{D}K}}V_{D}^{2}\right\}\;$$

where $K=V_{FB}-V_{G}+\frac{qN_{D}\varepsilon_{s}}{2C_{i}^{2}}=V_{T}-V_{G}+\frac{qN_{D}}{2\varepsilon_{s}}{\left(t_{s}+\frac{\varepsilon_{s}}{C_{i}}\right)}^{2}$.

The saturation voltage is calculated by evaluating $V_{D}$ for $dI_{DS}/dV_{D}=0$.

(A20) $$\left(t_{s}+\frac{\varepsilon_{s}}{C_{i}}-\sqrt{\frac{2\varepsilon_{s}K}{qN_{D}}}\right)=\frac{1}{2}\sqrt{\frac{2\varepsilon_{s}}{qN_{D}K}}V_{D,sat}$$

(A21) $$V_{D,sat}=2\sqrt{\frac{qN_{D}K}{2\varepsilon_{s}}}\left(t_{s}+\frac{\varepsilon_{s}}{C_{i}}\right)-2K\;\mathrm{Where},\;K=V_{T}-V_{G}+\frac{qN_{D}}{2\varepsilon_{s}}{\left(t_{s}+\frac{\varepsilon_{s}}{C_{i}}\right)}^{2}$$

#### Appendix A2.2.2. $V_{FB}<V_{G}$ (Bulk Conduction + Surface Conduction)

In the long-channel regime, when $V_{G}$ is raised higher than $V_{FB}$, there is no depletion region at $V_{D}=0$. The drain bias will only reduce the carrier density in the surface accumulation channel when $V_{D}<V_{G}-V_{FB}$. When $V_{D}=V_{G}-V_{FB}$, the surface accumulation channel is depleted at the drain end and the surface current is pinched off. When $V_{D}>V_{G}-V_{FB}$, the surface current is pinched off and the drain bias starts to reduce the carrier in the film.

##### $V_{D}\leq V_{G}-V_{FB}$

(A22) $$Q=Q_{s}+Q_{b}=C_{i}\left(V_{G}-V_{FB}-\Delta \psi \left(y\right)\right)+qN_{D}t_{S}$$

When $V_{D}\leq V_{G}-V_{FB}$, neither the surface nor the bulk are pinched off. Assuming a surface mobility, $\mu_{s}$, the surface current can be expressed as:

(A23) $$I_{s}=\frac{Z}{L}\mu_{s}\int_{0}^{V_{D}}\left(C_{i}(V_{G}-V_{FB}-\Delta \psi \left(y\right)\right)d\Delta \psi \left(y\right)=\frac{Z}{L}\mu_{s}C_{i}\left(\left(V_{G}-V_{FB}\right)V_{D}-\frac{1}{2}V_{D}^{2}\right)$$

The bulk current is:

(A24) $$I_{b}=\frac{Z}{L}\mu_{b}\int_{0}^{V_{D}}qN_{D}t_{S}d\Delta \psi \left(y\right)=\frac{Z}{L}\mu_{b}qN_{D}t_{S}V_{D}$$

The total current is:

(A25) $$I_{DS}=I_{s}+I_{b}=\frac{Z}{L}\left[\mu_{s}C_{i}\left(\left(V_{G}-V_{FB}\right)V_{D}-\frac{1}{2}V_{D}^{2}\right)+\mu_{b}qN_{D}t_{S}V_{D}\right]$$

Neither the surface nor the bulk are pinched off for Equation (A25), which means that $I_{DS}$ is still below the saturation limit. $V_{D,sat}$ can be obtained from the following condition:

(A26) $$\mu_{s}C_{i}\left(\left(V_{G}-V_{FB}\right)-V_{D,sat}\right)+\mu_{b}qN_{D}t_{S}=0$$

The resulting $V_{D,sat}$ is:

(A27) $$V_{D,sat}=\left(V_{G}-V_{FB}+\frac{\mu_{b}qN_{D}t_{S}}{\mu_{s}C_{i}}\right)>V_{G}-V_{FB}$$

This is not consistent with the assumption of V_D_ ≤ V_G_ −V_FB_. Therefore, the total current $I_{DS}$ will not reach the saturation current.

##### $V_{D}>V_{G}-V_{FB}$

When $V_{D}>V_{G}-V_{FB}$, we should divide the channel into two regions, where $\Delta \psi \left(y\right)<V_{G}-V_{FB}$ and $\Delta \psi \left(y\right)>V_{G}-V_{FB}$, because the portion of the surface potential that exceeds $V_{G}-V_{FB}$ will reduce the carrier density in the film.

For y where $\Delta \psi \left(y\right)<V_{G}-V_{FB}$:

(A28) $$Q=Q_{s}\left(V_{G},\;\Delta \psi \left(y\right)\right)+Q_{t}-Q_{D}\left(V_{G}=V_{FB},\Delta \psi \left(y\right)=0\right)\;=C_{i}\left(V_{G}-V_{FB}-\Delta \psi \left(y\right)\right)+qN_{D}t_{s}\;$$

For y where $\Delta \psi \left(y\right)>V_{G}-V_{FB}$:

(A29) $$Q=Q_{s}\left(V_{G},\Delta \psi \left(y\right)=V_{G}-V_{FB}\right)+Q_{t}-Q_{D}\left(V_{G}=V_{FB},\Delta \psi \left(y\right)=\Delta \psi \left(y\right)-\left(V_{G}-V_{FB}\right)\right)\;=qN_{D}\left(t_{s}-W_{D}\left(V_{FB},\Delta \psi \left(y\right)-\left(V_{G}-V_{FB}\right)\right)\right)\;$$

When $V_{D}>V_{G}-V_{FB}$, the surface potential will fully deplete the surface channel near the drain and reduce or deplete the bulk channel (depending on the magnitude of $V_{D}$). Therefore, we can assume that the surface channel is pinched off and the surface current will have a constant value of:

(A30) $$I_{s}=\frac{Z}{L}\mu_{s}C_{i}\frac{{\left(V_{G}-V_{FB}\right)}^{2}}{2}$$

On the other hand, carriers in the bulk of the channel are reduced by the component of the surface potential that exceeds $V_{G}-V_{FB}$:

(A31) $$Q_{b}=Q_{t}-Q_{D}\left(\Delta \psi \left(y\right)-V_{G}+V_{FB}\right)=qN_{D}\left(t_{s}-W_{D}\left(V_{FB},\Delta \psi \left(y\right)-V_{G}+V_{FB}\right)\right)$$

Therefore, the bulk current $I_{b}$ is:

(A32) $$I_{b}=\frac{qN_{D}Z}{L}\mu_{b}\left[\int_{0}^{V_{D}}t_{s}d\Delta \psi \left(y\right)-\int_{V_{G}-V_{FB}}^{V_{D}}W_{D}\left(V_{FB},\Delta \psi \left(y\right)-V_{G}+V_{FB}\right)d\Delta \psi \left(y\right)\right]$$

(A33) $$I_{b}=\frac{qN_{D}Z}{L}\mu_{b}\left[t_{s}V_{D}-\int_{V_{G}-V_{FB}}^{V_{D}}\left(\sqrt{\frac{2\varepsilon_{s}}{qN_{D}}\left(\Delta \psi \left(y\right)-V_{G}+V_{FB}\right)+{\left(\frac{\varepsilon_{s}}{C_{i}}\right)}^{2}}-\frac{\varepsilon_{s}}{C_{i}}\right)d\Delta \psi \left(y\right)\right]$$

(A34) $$I_{b}=\frac{qN_{D}Z}{L}\mu_{b}\left[\begin{array}{c} t_{s}V_{D}+\frac{\varepsilon_{s}}{C_{i}}(V_{D}-V_{G}+V_{FB})\\ -{\left[\sqrt{\frac{2\varepsilon_{s}}{qN_{D}}}\frac{2}{3}\;{\left(\Delta \psi \left(y\right)-V_{G}+V_{FB}+\frac{qN_{D}\varepsilon_{s}}{2C_{i}^{2}}\right)}^{\frac{3}{2}}\right]}_{V_{G}-V_{FB}}^{V_{D}} \end{array}\right]$$

(A35) $$I_{b}=\frac{qN_{D}Z}{L}\mu_{b}\left[\begin{array}{c} t_{s}V_{D}+\frac{\varepsilon_{s}}{C_{i}}(V_{D}-V_{G}+V_{FB})\\ -\sqrt{\frac{2\varepsilon_{s}}{qN_{D}}}\frac{2}{3}\;\left[{\left(V_{D}-V_{G}+V_{FB}+\frac{qN_{D}\varepsilon_{s}}{2C_{i}^{2}}\right)}^{\frac{3}{2}}-{\left(\frac{qN_{D}\varepsilon_{s}}{2C_{i}^{2}}\right)}^{\frac{3}{2}}\right] \end{array}\right]$$

Binomial approximation of the second order gives:

(A36) $$I_{b}=\frac{qN_{D}Z}{L}\mu_{b}\left\{\begin{array}{c} t_{s}V_{D}+\frac{\varepsilon_{s}}{C_{i}}(V_{D}-V_{G}+V_{FB})\\ -\frac{2}{3}\sqrt{\frac{2\varepsilon_{s}}{qN_{D}}}K^{\frac{3}{2}}\left(\begin{array}{c} 1+\frac{3}{2}\frac{\left(V_{D}-V_{G}+V_{FB}\right)}{K}+\\ \frac{1}{2}\frac{3}{2}\;\left(\frac{3}{2}-1\right){\left(\frac{\left(V_{D}-V_{G}+V_{FB}\right)}{K}\right)}^{2}-1 \end{array}\right) \end{array}\right\}$$

Here:

$$K\equiv \frac{qN_{D}\varepsilon_{s}}{2C_{i}^{2}}$$

Then:

(A37) $$I_{b}=\frac{qN_{D}Z}{L}\mu_{b}\left\{\begin{array}{c} t_{s}V_{D}+\frac{\varepsilon_{s}}{C_{i}}(V_{D}-V_{G}+V_{FB})\\ -\frac{2}{3}\sqrt{\frac{2\varepsilon_{s}}{qN_{D}}}\;K^{\frac{3}{2}}\;\left(\frac{3}{2}\frac{\left(V_{D}-V_{G}+V_{FB}\right)}{K}+\frac{1}{4}\frac{3}{2}\;{\left(\frac{\left(V_{D}-V_{G}+V_{FB}\right)}{K}\right)}^{2}\right) \end{array}\right\}$$

(A38) $$I_{b}=\frac{qN_{D}Z}{L}\mu_{b}\left\{\begin{array}{c} t_{s}V_{D}+\left(\frac{\varepsilon_{s}}{C_{i}}-\sqrt{\frac{2\varepsilon_{s}K}{qN_{D}}}\right)\;\left(V_{D}-V_{G}+V_{FB}\right)\\ -\frac{1}{4}\sqrt{\frac{2\varepsilon_{s}}{qN_{D}K}}{\left(V_{D}-V_{G}+V_{FB}\right)}^{2} \end{array}\right\}$$

Replacing $K\equiv \frac{qN_{D}\varepsilon_{s}}{2C_{i}^{2}}$ results in $\frac{\varepsilon_{s}}{C_{i}}-\sqrt{\frac{2\varepsilon_{s}K}{qN_{D}}}=0$ and the equation becomes:

(A39) $$I_{b}=\frac{qN_{D}Z}{L}\mu_{b}\left\{t_{s}V_{D}-\frac{C_{i}}{2qN_{D}}{\left(V_{D}-V_{G}+V_{FB}\right)}^{2}\right\}\;$$

Therefore, the total current is:

(A40) $$I_{DS}=I_{s}+I_{b}=\frac{Z}{L}\mu_{s}C_{i}\frac{{\left(V_{G}-V_{FB}\right)}^{2}}{2}+\frac{qN_{D}Z}{L}\mu_{b}\left\{t_{s}V_{D}-\frac{C_{i}}{2qN_{D}}{\left(V_{D}-V_{G}+V_{FB}\right)}^{2}\right\}$$

The saturation voltage can then be found to be:

(A41) $$t_{s}-\frac{C_{i}}{qN_{D}}(V_{D,sat}-V_{G}+V_{FB})=0$$

(A42) $$V_{D,sat}=V_{G}-V_{FB}+\frac{qN_{D}}{C_{i}}t_{s}$$

### Appendix A2.3. Parameters for Figure 2

The following is the list of parameters used to generate plots in Figure 2.

**Table A9:** List of parameters used in the plots in Figure 2.

Symbol	Parameter	Value	Notes
$N_{D}$	Doping concentration of the semiconductor	1 × 10^17^cm^−3^	Same as Figure 1
$q\phi_{m}$	Work function of metal	5.1 eV	Same as Figure 1
$q\chi_{i}$	Electron affinity of the insulator	2.58 eV	
$d_{\;}$	Thickness of the gate insulator	50 nm	
$q\chi_{s}$	Electron affinity of the semiconductor	4.3 eV	Same as Figure 1
$q\phi_{s}$	Work function of semiconductor	4.76 eV	Same as Figure 1
$T_{\;}$	Temperature	300 K	Same as Figure 1
$\varepsilon_{s}$	Permittivity of the semiconductor	8.5$\varepsilon_{0}$	Same as Figure 1
$m_{e}$	Effective electron mass of the semiconductor	0.19$m_{0}$	Same as Figure 1
$m_{h}$	Effective hole mass of the semiconductor	0.21$m_{0}$	Same as Figure 1
$E_{g}$	Energy bandgap	3.35 eV	Same as Figure 1
$\mu_{b}$	Electron mobility of the bulk semiconductor	10 cm^2^/Vs	
$\mu_{s}$	Electron mobility of the accumulation channel	10 cm^2^/Vs	
$d_{33}$	Piezoelectric moduli in z-axis direction	12.4 pC/N	Same as Figure 1
F/A	Applied pressure to the junction	30 MPa	
$\delta_{s}$	Thickness of the piezoelectric charge	0.5 nm	Assumption
r	Reduction ratio of the back surface piezoelectric charge	0.5	Assumption
